# Local Energy Landscape Drives Long-Range Exciton Diffusion in Two-Dimensional Halide Perovskite Semiconductors

**DOI:** 10.1021/acs.jpclett.1c00823

**Published:** 2021-04-20

**Authors:** Alan Baldwin, Géraud Delport, Kai Leng, Rosemonde Chahbazian, Krzysztof Galkowski, Kian Ping Loh, Samuel D. Stranks

**Affiliations:** † Cavendish Laboratory, University of Cambridge, JJ Thomson Avenue, Cambridge CB3 0HE, U.K.; ‡ Department of Chemical Engineering & Biotechnology, University of Cambridge, Philippa Fawcett Drive, Cambridge CB3 0AS, U.K.; § Department of Applied Physics, The Hong Kong Polytechnic University, Hung Hom, Kowloon, Hong Kong, China; ∥ Department of Chemistry, National University of Singapore, Singapore, Singapore; ⊥ Institute of Physics, Faculty of Physics, Astronomy and Informatics, Nicolaus Copernicus University, Fifth Grudziadzka St., 87-100 Toruń, Poland

## Abstract

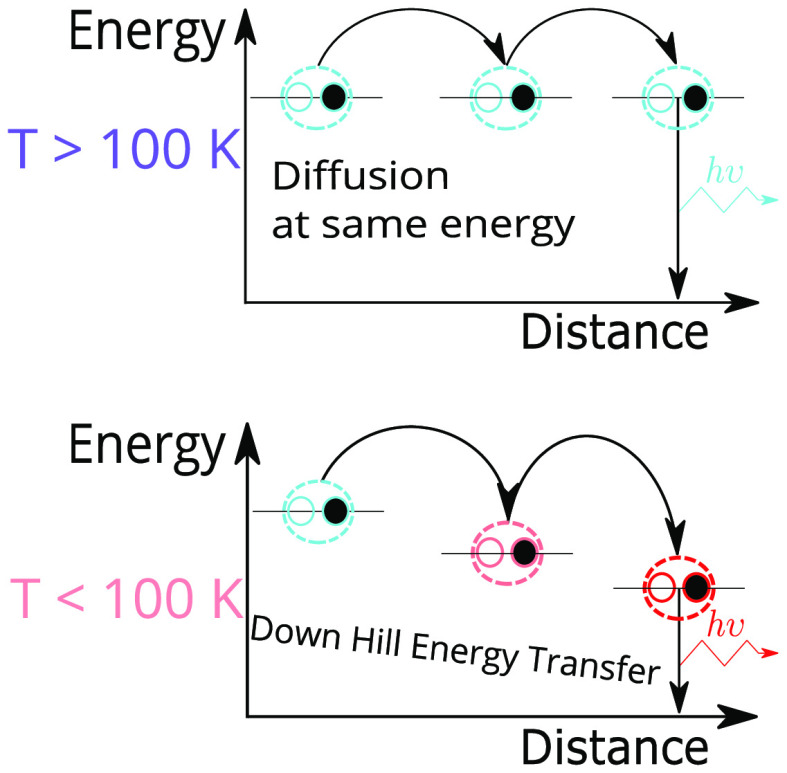

Halide perovskites are versatile semiconductors with applications including photovoltaics and light-emitting devices, having modular optoelectronic properties realizable through composition and dimensionality tuning. Layered Ruddlesden–Popper perovskites are particularly interesting due to their unique 2D character and charge carrier dynamics. However, long-range energy transport through exciton diffusion in these materials is not understood or realized. Here, local time-resolved luminescence mapping techniques are employed to visualize exciton transport in exfoliated flakes of the BA_2_MA_*n*–1_Pb_*n*_I_3*n*+1_ perovskite family. Two distinct transport regimes are uncovered, depending on the temperature range. Above 100 K, diffusion is mediated by thermally activated hopping processes between localized states. At lower temperatures, a nonuniform energy landscape emerges in which transport is dominated by downhill energy transfer to lower-energy states, leading to long-range transport over hundreds of nanometers. Efficient, long-range, and switchable downhill transfer offers exciting possibilities for controlled directional long-range transport in these 2D materials for new applications.

Halide perovskites have intrigued the semiconductor community for the past decade, challenging the established understanding that defect concentrations in semiconductors must be minimized at all costs for high device performance,^1^ which typically requires complex fabrication methods. Remarkably, the perovskite structure offers the possibility to tune the crystal dimensionality and the inherent photophysical properties, for example, by changing the length and nature of the A-site cation out of a large library of compatible molecules.^2^ Ruddlesden–Popper perovskites (RPPs) hold a special place in the perovskite family due to their unique 2D character (Figure 1a) and their enhanced stability with respect to conventional 3D perovskites in the atmosphere and under illumination.^3,4^ These RPP materials take the general formula S_2_A_*n*–1_B_*n*_X_3*n*+1_, where S represents a large organic spacer cation, A is a small monovalent cation, B is a divalent metal, and X is a halide anion. Structurally, they are composed of corner-sharing BX_6_ octahedra quasi-2D layers, with *n* representing the number of BX_6_ planes, with the layers separated by bulky S molecules.^5−7^ High-quality single-crystal RPPs can be fabricated and mechanically exfoliated to yield any desired number of layers,^6,8^ paving the way for ultrathin optoelectronic devices.^9^ The two-dimensionality of each of the layers coupled with the low dielectric constant of the spacer molecules results in charge dynamics which are dominated by strongly bound excitons.^5,10−12^ The remarkable photophysical properties of these RPPs have been utilized in various domains such as lasers^13^ and LEDs^14^ and in efficient, stable perovskite solar cells.^15^ In particular, long-lived and highly diffusive excitons can be generated in such RPPs, while their emission spectra can be greatly modified due to local low-energy states^16^ or the presence of *n* RPP domains^17^ within the same sample. The study of the charge carrier diffusion in such RPPs is a complex but essential goal for ultimately attaining fine control over energy transport in these materials and associated devices. In this context, recent studies have started to disentangle the influence of photon recycling,^18^ phonons,^19^ or shallow trap states,^20^ paving the way toward a better understanding of the global charge carrier transport mechanisms in such RPPs. Nevertheless, many aspects related to exciton recombination and transport, particularly on the local scale, and how local physicochemical properties impact these phenomena remain to be investigated.

**Figure 1:**
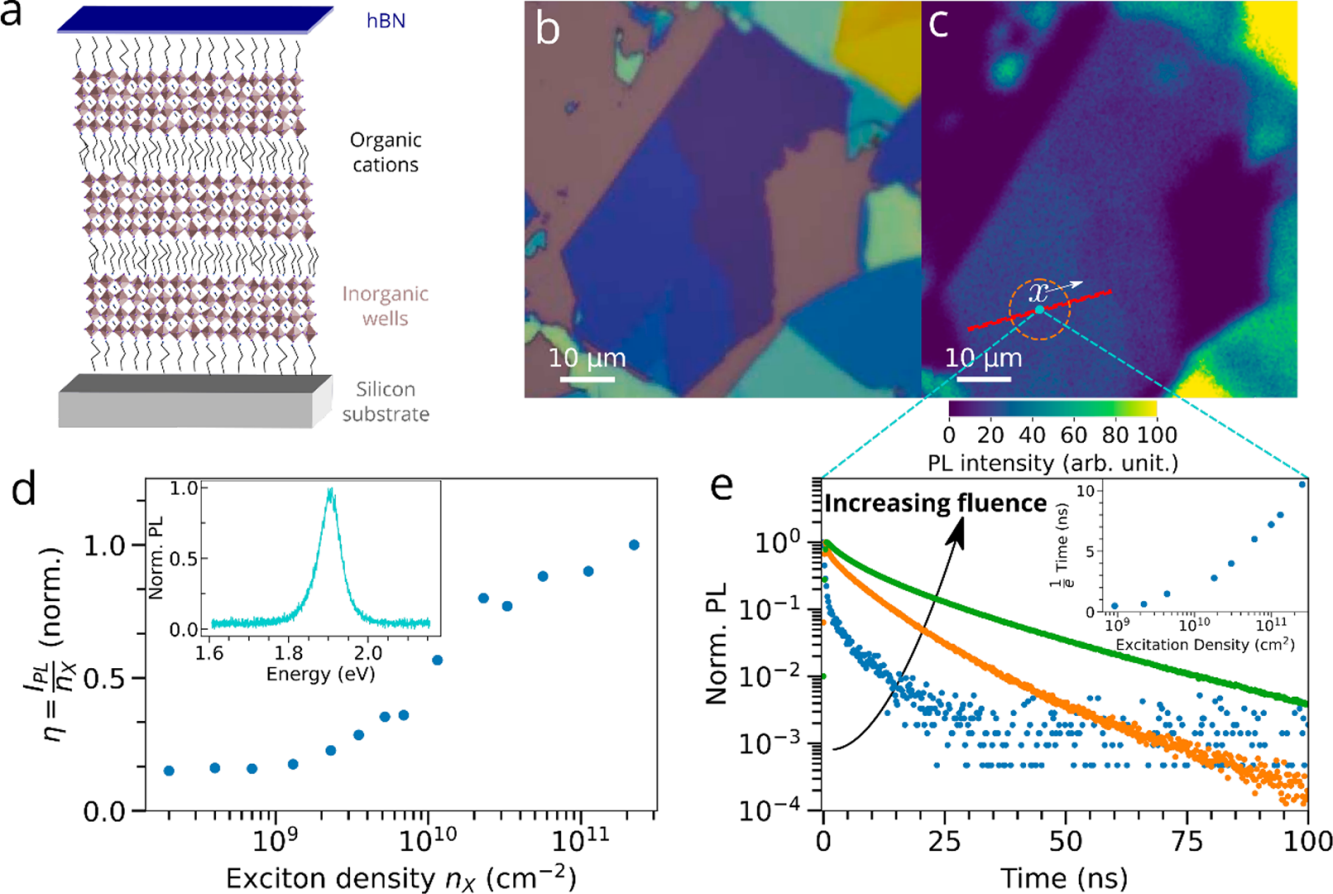
Local photoluminescence properties of the studied BA_2_MA_3_Pb_4_I_13_*n* = 4 RRP flake at room temperature. (a) Schematic representation of a few-layer *n* = 4 RRP flake. (b) Optical reflection and (c) PL intensity (excitation at 510 nm) images. The red strip in c highlights the region of interest over which transport is subsequently analyzed, with spatial parameter *x* defined as spanning from the center of the region. (d) Evolution of the normalized PL efficiency η as a function of the injected exciton density, *n*_*x*_, with η defined as the ratio of the number of emitted photons (PL intensity, *I*_PL_) to the number of injected excitons *n*_*x*_, arbitrarily normalized to 1 for clarity. (Inset) Normalized PL spectrum at the same location. (e) Time-resolved PL decays for increasing injected exciton densities in the center of the region of interest highlighted in c, with exciton density increasing from 9.2 × 10^8^ cm^–2^ (blue) to 3.0 × 10^10^ cm^2^ (orange) to 2.5 × 10^11^ cm^–2^ (green) for emitted wavelengths shorter than ∼800 nm. (Inset) 1/*e* time for TRPL decays as a function of the excitation density.

Here, we employ time-resolved photoluminescence microscopy^21−23^ (TRPL), coupled to a helium cryostat, to visualize the local charge carrier dynamics and diffusion properties as a function of temperature in BA_2_MA_*n*–1_Pb*_n_*I_3*n*+1_ (BA = butylammonium, MA = methylammonium) flakes. (See SI Sections I–IV and Figure S1 for a description of the customized confocal photoluminescence setup.) We focus on the *n* = 4 system, with similar results obtained for *n* = 2, with the flakes having been exfoliated from macroscopic single crystals. The exciton binding energy in such *n* = 4 (or *n* = 2) perovskites is on the order of hundreds of millielectron volts,^10^ implying that the carriers form a majority of excitons under all but one of the experimental conditions used in our study. (See SI Section V and Figure S2 for details.) For temperatures in the range of 300–100 K, we observe diffusive transport in which exciton–phonon coupling plays a crucial role, resulting in a thermally activated hopping process through localized states, which are also responsible for the nonradiative decay of these excitons. At temperatures below 100 K, such hopping processes become inefficient and local energy gradients take precedence over exciton–phonon coupling, leading to a transport regime driven by local energy gradients across a varying band gap landscape originating from local phase or structural variations. Such energy gradients lead to exciton transport on length scales of up to 600 nm (SI Section VI) associated with strong luminescence from low-energy sites in the flakes. This study reveals that long-range directional energy transport is possible if local energy landscapes can be controlled and exploited, paving the way for new devices based on 2D materials including transistors, light-emitting devices, and photodetectors.

We focus our study on the exciton dynamics in *n* = 4 exfoliated thin RPP crystals deposited on a silicon substrate and encapsulated with a hexagonal boron nitride (h-BN) layer to ensure environmental and illumination stability.^9^ (See SI Section I for details and Figure 1a for the schematic structure of the *n* = 4 sample.) We also observe similar results for *n* = 2 crystals (SI Section XIII) to those reported here. An optical image of one of the flakes of interest is shown in Figure 1b with a thickness of ∼9 stacked quantum wells (∼30 nm thickness), based on the optical contrast^6^ of the flake and on previous studies^9^ (details in SI Section VII and Figure S3), with PL (Figure 1d) and XRD (SI Section VIII and Figure S4) measurements suggesting that the flakes are highly phase-pure. Such thin flakes maximize our chances to separate the exciton diffusion process from other physical effects to accurately capture the thermal evolution of the excitonic diffusion and recombination processes. Specifically, photonic effects (reabsorption, recycling, or lateral waveguiding effects^24−26^) will be negligible in both the vertical and lateral directions, and phonon-mediated phases transitions are forbidden in such thin flakes.^27^

Figure 1c displays the photoluminescence (PL) intensity map of the flake of interest. The local PL spectrum of this flake (Figure 1d inset) is centered at ∼1.9 eV, as expected for a high-quality pure-phase *n* = 4 flake,^16^ suggesting no other *n* phases are present in that region (SI Section VIII and Figure S4). We first measure the local PL properties in the region of interest (Figure 1c) in different excitation regimes with a 510 nm pulsed laser diode. (See SI Section IX for details and Figures S5 and S6 for further data.) In Figure 1d, we show the PL intensity as a function of excitation density *n*_*x*_, weighted by the excitation density *n*_*x*_, to provide a relative PL efficiency η (i.e., normalized number of emitted photons per injected exciton; see SI Section X for further data and Figure S7 for the absolute PL intensity). We observe that this curve forms an S shape composed of two plateaus separated by a growth phase, which can be ascribed to different regimes of trap state filling.^28^ Below *n*_*x*_ ≈ 2 × 10^9^ cm^–2^, the relative PL efficiency remains constant, indicating that the trap-filling process is inefficient in this range of exciton densities. This is explained by the fact that the concentration of available traps remains much larger than that of injected excitons across this excitation regime. Above this *n*_*x*_ value, η increases significantly as the PL intensity increases superlinearly with respect to the exciton density. Across this regime, the exciton density is large enough to fill a non-negligible proportion of traps,^7^ leading to an increase in the PL efficiency. This trap filling occurs up to a saturation of this effect at *n*_*x*_ ≈ 3 × 10^10^ cm^–2^, above which η again remains constant with increasing excitation density and a large proportion of excitons are recombining radiatively. Similar observations can be made when considering time-resolved PL (TRPL) decays as a function of the exciton density (Figures 1e and 1e inset, S8, and S9). In the low exciton density regime (9.2 × 10^8^ cm^–2^, blue data) corresponding to the first plateau, the TRPL curve exhibits a rapid decay, with a PL lifetime of 0.5 ns defined as the time to fall to 1/*e* of the initial intensity,^23^ consistent with the fast nonradiative decay of trapped excitons as the primary recombination channel. Above this first plateau, the TRPL lifetime increases with increasing excitation density (3.0 × 10^10^ cm^–2^, orange data) until the second plateau is reached (Figures 1e inset and S9). At this second plateau (2.50 × 10^11^ cm^–2^, green data), we observe a quasi-monoexponential decay with a 1/*e* PL lifetime of ∼11 ns, which is consistent with classical first-order exciton recombination kinetics dominated by a radiative rate.^7^

We now use the same TRPL microscope setup to investigate the impact of this trap-filling process on exciton transport. In this configuration, the sample is excited in the same local region at time *t* = 0 at a fixed position (*x* = 0) with a Gaussian-shaped laser pulse (cf. blue circle Figure 1c), with the PL subsequently collected as a function of time (*t*) at different spatial points (*x*) away from the excitation spot. (See Figure S1 for the setup.) The resulting spectrally integrated spatial PL profiles, from the region highlighted in Figure 2a, are displayed as a function of time in Figure 2b in the low excitation density regime (*n*_*x*_ ≈ 1.0 × 10^8^ cm^–2^), with the increase in the standard deviation of the Gaussian spread σ(*t*) with time consistent with excited species spreading laterally after excitation. (See SI Section XII and Figures S11 and S12 for Gaussian profiles and fits at selected times.) We find a linear relationship between the quantity [σ^2^(*t*) – σ^2^(0)] and *t* (Figure 2c), indicating that the spatial broadening is due to classical exciton diffusion^23,29^ and can be related to the exciton diffusion coefficient *D* using the formula^23,29,30^
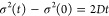
1Linear fits to the data in Figure 2c with eq 1 yield a diffusion coefficient of 0.018 cm^–2^ s^–1^ for *n*_*x*_*=* 1.0 × 10^8^ cm ^–2^ (Figure 2c inset). At this low exciton density, we expect exciton trapping to dominate on the basis of the earlier PL efficiencies, η. At higher exciton densities corresponding to the beginning of the trap saturation regime (*n*_*x*_ = 9.2 × 10^8^ cm^–2^), the fitted diffusion coefficient increases slightly to 0.021 cm^–2^ s^–1^ and then increases significantly to 0.096 cm^–2^ s^–1^ at even higher exciton densities (*n*_*x*_ = 1.3 × 10^10^ cm^–2^) in which a large proportion of the traps are saturated. We note that there is no significant deviation from the linear evolution of [σ^2^(*t*) – σ^2^(0)] across these excitation densities for the accessible time scales, suggesting that nonlinear effects such as exciton–exciton annihilation do not influence the exciton dynamics. These measurements reveal that the local trap states not only limit the PL efficiency but also limit exciton diffusion in these RPPs systems, consistent with other 2D semiconductors.^20,31^ Similar behavior is observed for an *n* = 2 sample (SI Section XIII and Figures S13–S16).

**Figure 2:**
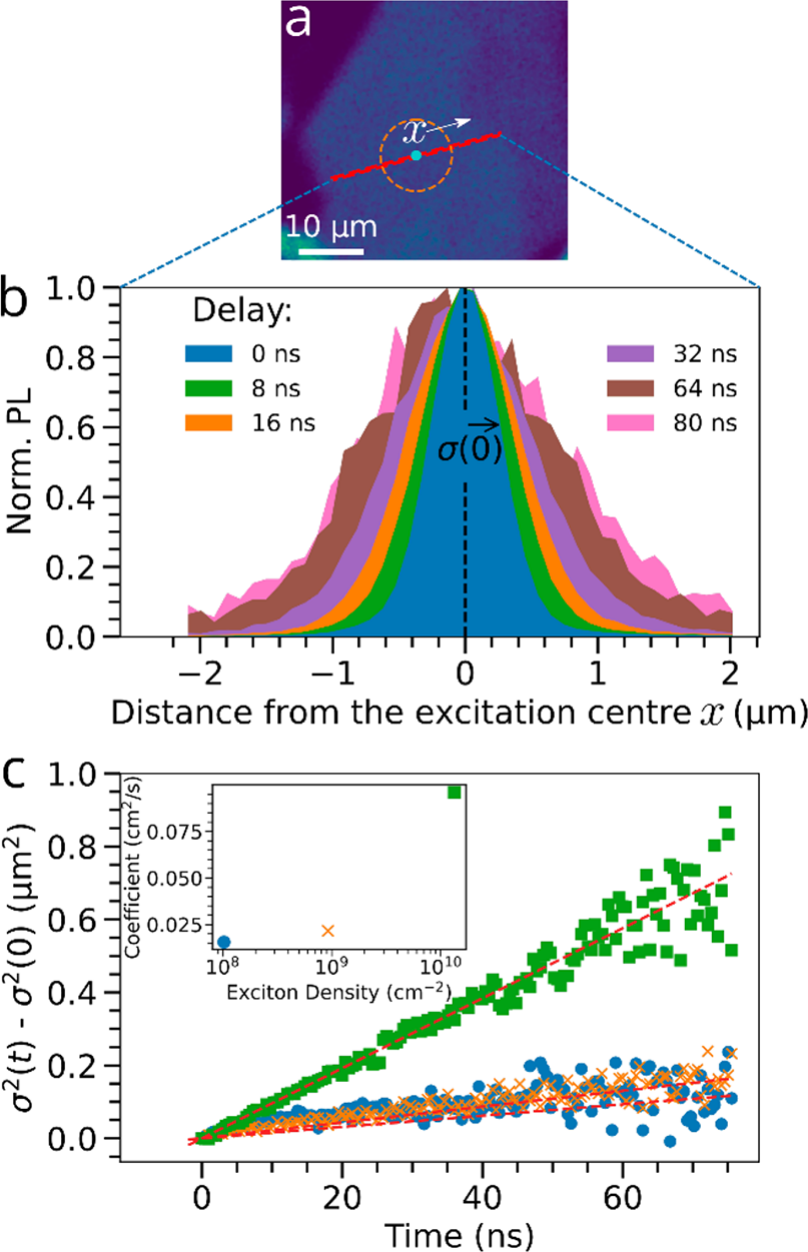
Local spatially resolved diffusion measurements in a BA_2_MA_3_Pb_4_I_13_ (*n* = 4) RRP flake at different fluences. (a) PL map highlighting the region of interest. (b) Selected spatial PL profiles, normalized, at different times after excitation by the laser pulse, localized at *x* = 0 (center of the red line in Figure 2a). (c) Spreading profiles showing the temporal evolution of the squared broadening quantities σ^2^(*t*) – σ^2^(0) of the spatial profiles for the flake at room temperature in the region of interest at three different densities of injected excitons. (Inset) Diffusion coefficients extracted from fits to the data with eq 1. To clearly visualize the spreading effect, the profiles shown in (b) were obtained with a 0.8 NA 100× objective lens (generating a narrower initial exciton distribution), whereas all other data throughout, including those in panel c, were taken with a 0.4 NA 10× objective lens which is more suitable for the cryogenic measurements and does not alter the diffusion measurements (SI Section XI and Figure S10).

To further understand the exciton dynamics and transport, we performed temperature-dependent studies on the same region of the *n* = 4 flake. (See SI Section XIV for further PL data.) We show local PL spectra in Figure 3a as a function of temperature. (See Figure S17 for spectra at other temperatures and Figure S18 for the integrated PL intensity.) The position of the main PL resonance, which we refer to herein as the high-energy (HE) peak, does not shift significantly from ∼1.9 eV upon decreasing temperature from 300 to 80 K. This lack of shift is consistent with the strong influence of phonons on the exciton properties^11,32,33^ that counterbalances the usual PL red shift in 3D perovskites attributed to the thermal contraction of the crystal within this temperature range^34,35^ rather than other effects such as self-trapped excitons.^32^ The red-shift behavior is recovered over the range of 120 to 10 K where the contribution of optical phonons should be negligible. Indeed, the lowest optical phonon mode energy in similar systems has been reported to be ∼10 meV,^35−37^ corresponding to threshold temperatures of around ∼120 K. Further evidence for the influence of phonons on the exciton properties is seen in the decrease in the PL resonance width of the HE peak from ∼99 meV at 300 K to 22 meV at 100 K (Figure S19).^32,34^

**Figure 3:**
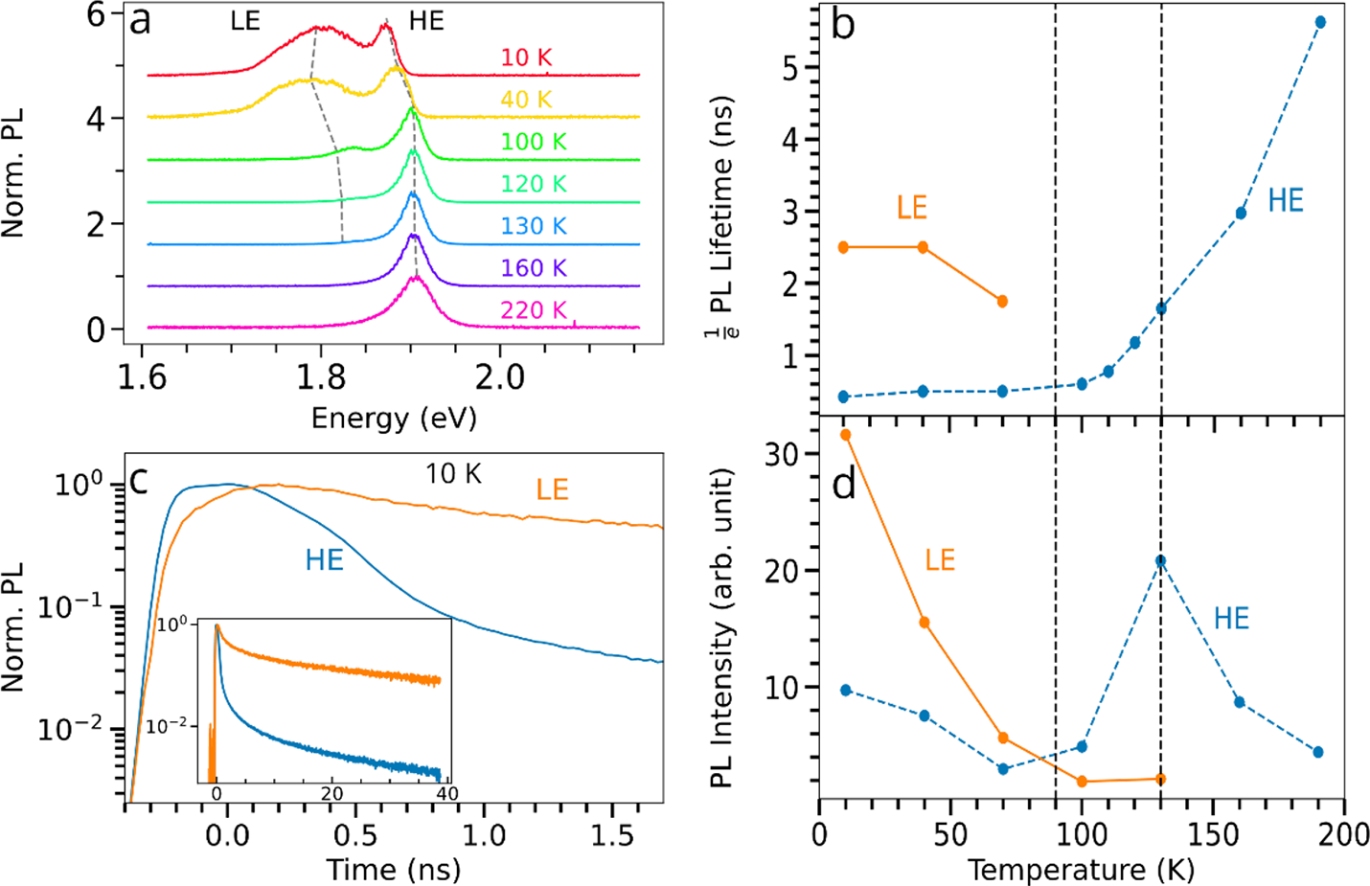
Temperature-dependent PL spectra and exciton dynamics in a BA_2_MA_3_Pb_4_I_13_RRP flake. Measurements were taken in the center of the region of interest investigated earlier. (a) PL spectra showing the evolution of the high-energy (HE) peak and the emergence of the low-energy (LE) peak at low temperatures, with excitation at 510 nm. Spectra are normalized to the maximum value of each spectrum and vertically offset for clarity. Gray dotted lines tracking the peak centers are guides to the eye. Spectrally selected (b) 1/*e* lifetimes of the LE and HE components. (c) Spectrally selected TRPL decays corresponding to the HE and the LE resonance at 10 K, exhibiting the population-transfer process. (Inset) Corresponding decay on the longer time scale. (d) Spectrally selected PL intensities as a function of temperature. Measurements were performed with an excitation density of 9.2 × 10^8^ cm^–2^. Spectral selection is achieved using appropriate band-pass filters (SI Section XVI and Figure S22).

To further understand the exciton dynamics, we analyzed the evolution of the TRPL decays as a function of temperature. (See Figure S20 for all decays.) The 1/*e* PL lifetime in the HE peak decreases significantly from ∼6 ns at 190 K to 2.9 ns at 100 K with an exciton density of 9.2 × 10^8^ cm^–2^ (Figure 3b). We note that such relatively short PL lifetimes are to be expected for excitonic semiconductors which possess large oscillator strengths. The general decrease in the PL lifetime with decreasing temperature can again, in part, be attributed to a freezing out of phonons, either by the lack of exciton–phonon coupling at low temperatures preventing excitons from escaping local traps states or, by analogy with MoSe_2_^38^ or GaAs quantum wells,^39^ a lack of phonons that otherwise push excitons away from the bottom of the band and thus prevent recombination until they return. Interestingly, this decrease in the PL lifetime is first accompanied by an increase in the PL intensity down to 130 K (Figure 3d), but below 130 K the PL intensity decreases significantly. This drop in PL intensity at ∼130 K occurs concomitantly with the emergence (Figure 3a) and rapid increase in intensity (Figure 3d) of a second, broader PL resonance at ∼1.8 eV, referred to hereafter as the low-energy (LE) resonance. This LE resonance indicates the presence of radiative states situated energetically below the classical excited-state continuum. Indeed, LE resonances have been previously reported in 2D perovskites and often attributed either to self-trapped excitons^40−44^ (STE) or defect-assisted emission^45^ (DAE). To our knowledge, there is no strong evidence of self-trapped exciton properties in the BA-based RPPs family,^46^ while such properties are regularly observed in RPP families based on specific organic cations leading to distortions of the perovskite lattice.^47^ In addition, the STE PL intensity is often maximal at an intermediary temperature of around 100 K,^48^ while our LE resonance is maximal at 10 K (Figure 3a). Finally, we show in Figure S21 (SI Section XV) that the LE resonance saturates at a high excitation intensity in comparison to the HE one, which suggests a local and extrinsic emission process rather than an intrinsic process that would occur evenly across the sample and would saturate. We will therefore mostly consider the DAE hypothesis in the following analyses and simulations. Nevertheless, many of our observations would also be compatible with a hybrid (STE/DAE) situation that has been reported recently^43^ in which the STE effects are heterogeneously triggered across the sample due to the presence of extrinsic local features. On the basis of the literature^40−44^ and our spectroscopic measurements including the ability to saturate the states, we do not believe that the LE states result from a phase change within the material. Unambiguous assignment of the precise nature of the LE states will require further studies that are beyond the scope of the present work.

In many lead halide perovskites materials, the LE emission has been associated with local impurity phases such as excess iodine precursors^45,49^ and is generally only readily apparent at low temperature.^44,45,50,51^ We note that the nature of these localized radiative states appears to be distinct from the nonradiative trap states discussed above, consistent with a general increase in the total PL intensity (LE + HE) observed between 200 and 10 K (Figures 3d and S18). We cannot definitively rule out the presence of the LE states above 100 K; however, if the LE states are present, then their impact should be negligible, as attested to by the PL spectra showing only minor tails in the LE region above 100 K (Figures 3a and S17).

The decrease in intensity of the HE peak together with the rise in intensity of the LE peak at low temperature hints at a transfer of exciton population from the HE to LE states,^16^ and we will herein refer to this as downhill energy transfer. This transfer effect is evident by considering the spectrally resolved PL dynamics, achieved using appropriate band-pass filters (SI Section XVI and Figure S22), at a given temperature, exemplified in Figure 3c at 10 K (SI Section XVII and Figure S23 for other temperatures). Within the first nanosecond after the excitation pulse, we observe both a fast decline of the HE component and a rise of the LE component with a rise time of ∼0.5 ns (time between the maximum of the HE component and the maximum of the LE component), longer than the setup instrument response of ∼385 ps (SI Section XVIII and Figure S24). After another few hundred picoseconds, the LE component starts declining but at a reduced rate compared to the HE signal. The PL decay of the LE component extends over ∼40 ns (Figure 3c), suggesting that once the excitons reach these LE states, their recombination is slower than in the HE states.

We now consider the temperature-dependent transport properties and the impact of this energy-transfer process on these properties. We start by analyzing the diffusion properties associated with the HE peak as a function of temperature at a low exciton density of *n*_*x*_ = 9.2 × 10^8^ cm^–2^. We limit our measurements to temperatures below 200 K to maintain a low pressure without disturbing the measurements with the vibrating vacuum cryopump (SI Sections II–IV). We find that the diffusion coefficient decreases significantly from 0.03 cm^2^ s^–1^ at 200 K to a negligible value (<0.001 cm^2^ s^–1^) below 100 K. (See Figure 4c and an example fit at 160 K in Figure 4b; see SI Section XIX and Figures S25–S28 for Gaussian profiles and resulting spreading curves at different temperatures.) This suggests that the excitons are progressively less likely to transfer through the HE sites with decreasing temperature. We note that PL spectra from the studied region, taken before and after each diffusion measurement, show no appreciable change in the HE resonance at any temperature and only small shifts in the LE resonance at 100 and 130 K, ruling out structural changes or laser-induced damage occurring during the measurements and influencing the results (SI Section XX and Figure S29).

**Figure 4:**
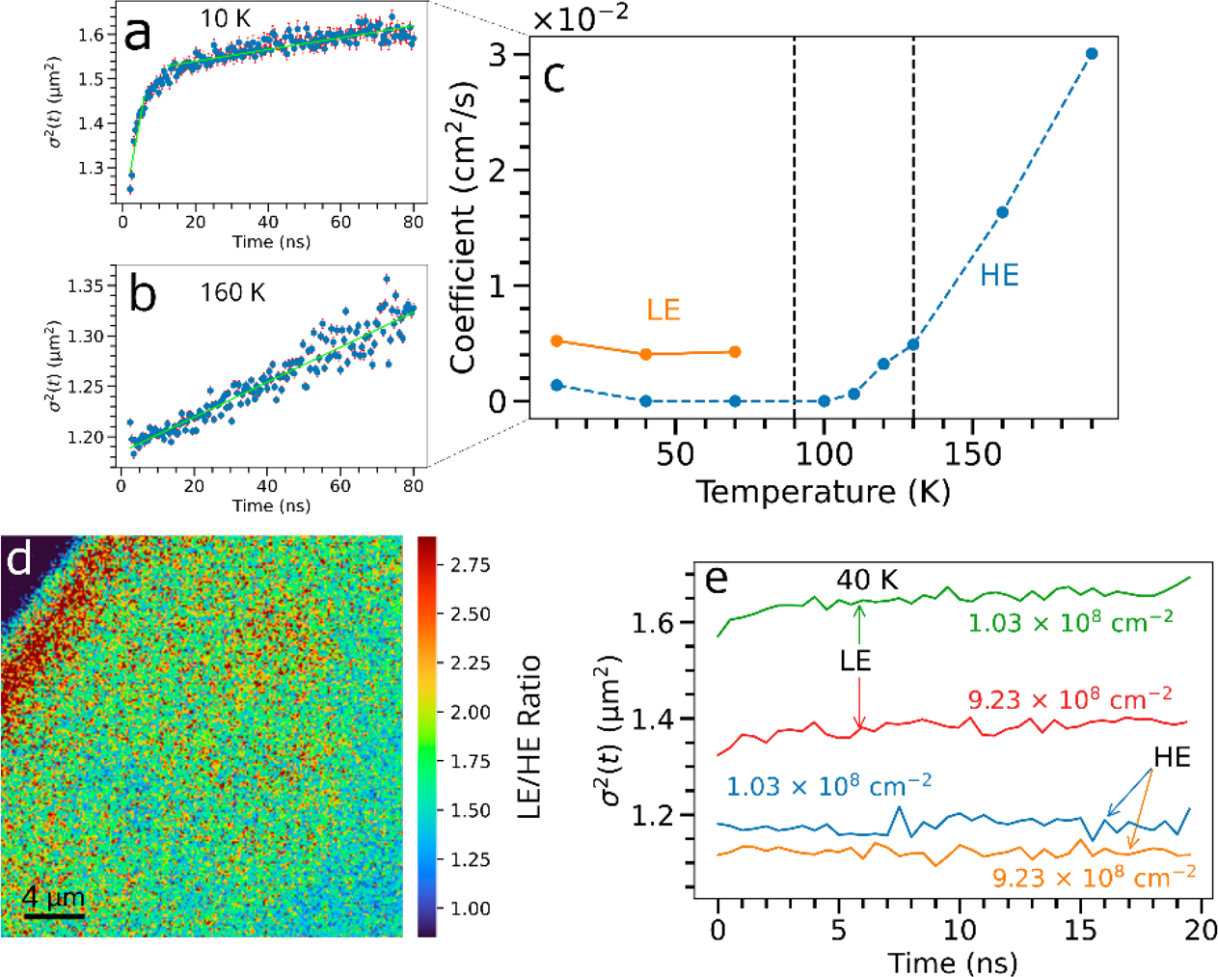
Temperature-dependent transport properties in a BA_2_MA_3_Pb_4_I_13_RRP flake. Spreading profiles at (a) 10 K and (b) 160 K with an excitation density of 9.2 × 10^8^ cm^–2^. (c) Effective spreading coefficients extracted from fitting the underlying spreading profiles to eq 1, exemplified by the linear fits in green in a and b (Figures S26 and S28). For the LE components, only the initial fast spreading coefficients are shown. (d) Ratio of normalized confocal PL maps at 10 K with excitation at 510 nm of the flake allowing the distinction of the regions containing LE and HE peaks, obtained using band-pass filters. (e) Spreading profiles at 40 K for different injected exciton densities with LE and HE peaks distinguished using the band-pass filters (SI Section XVI and Figure S22).

To consider the transport associated with the LE and HE sites, we show in Figure 4d a map, which is a ratio of PL maps taken using appropriate band-pass filters (SI Section XVI and Figure S22), of the region of interest at 10 K, showing a spatial distribution of these LE and HE sites. Fluence-dependent measurements show that the emission from the LE states saturates when the excitation density is on the order of ∼9 × 10^8^ cm^–2^ (Figure S21), indicating that a significant portion of the created excitons would have been unable to access these LE states. This is likely due to a lower concentration of LE regions across the flake, together with slower recombination, compared to the HE regions. This result shows that the spatial energy landscape is composed of many HE-only domains, sparsely interspersed with LE subdomains. Because these LE and HE states are dispersed across the sample, we now consider whether such an exciton transfer system observed can promote a lateral motion of the excitons beyond the classical diffusive process. We show the extracted exciton-transport coefficient corresponding to the LE and HE peaks in Figure 4c, again at a low exciton density of 9.2 × 10^8^ cm^–2^. (See Figure 4a for an example of the spreading observed for the LE peak at 10 K.) A very slow spatial spreading of the PL profile is observed for the HE resonance, comparable to the diffusion coefficient measured just above 100 K. However, the LE band exhibits an initial fast spreading of the spatial PL profile, on the order of 1 ns, followed by a regime over the next ∼80 ns during which negligible spreading occurs. Further fluence-dependent measurements (Figure 4e) reveal that the transport contribution associated with the LE bands saturates at a higher exciton density, consistent with the saturation of the PL from these states. These observations indicate that an efficient lateral motion of the excitons occurs on the same nanosecond time scale as the HE-to-LE population transfer discussed earlier. We propose that the two effects are connected and that the large energy gradient between adjacent HE and LE regions causes excitons to rapidly drift toward the LE states, dispersed throughout the flake, through the downhill energy-transfer mechanism introduced earlier (Figure 5b). With this in mind, we propose that *D*, the diffusion coefficient, be renamed as the spreading coefficient (cm^–2^ s^–1^) to account for the fact that diffusion is not the only transport mechanism that will affect the rate of spreading of the PL profile (Figure 4c).

**Figure 5:**
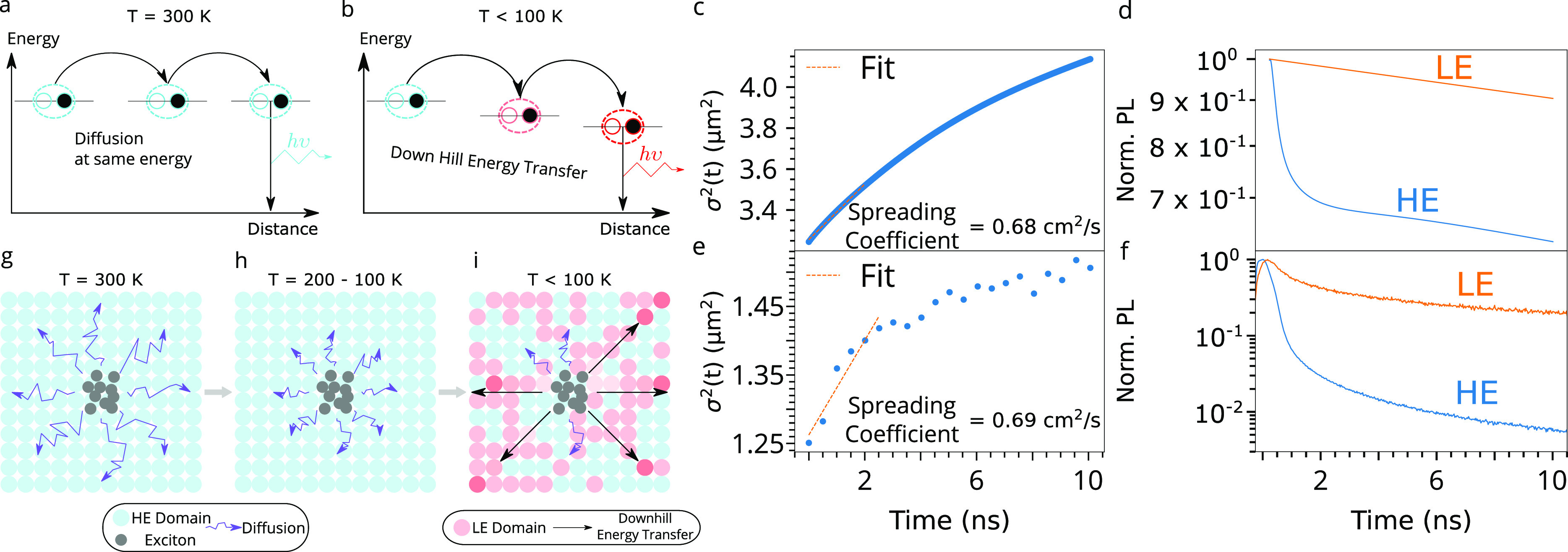
Understanding energy-transfer mechanisms in a BA_2_MA_3_Pb_4_I_13_RRP flake at varying temperatures. (a) At room temperature, diffusion mediated by a thermally activated hopping processes is the dominant exciton-transport mechanism. (b) Downhill energy-transfer transport to lower-energy sites at lower temperature. Modeled (c) spreading curve and (d) log-scaled TRPL decays. Experimental (e) spreading curve and (f) TRPL decays from the actual flake at an exciton density of 9.2 × 10^8^ cm^–2^. (g–i) Schematics to represent the proposed transport model over the studied temperature ranges. (g) Dominant diffusive transport at room temperature. (h) As the temperature is lowered, the diffusion process becomes less efficient. (i) At *T* < 100 K, the LE states emerge and downhill energy transfer becomes the dominant transport mechanism. The lengths of the diffusion and downhill energy-transfer indicators are indicative of the rates of these processes. The output is from simulations of our proposed model (eq 2) compared to experiment. See SI Section XXI for the parameters that were used.

To validate these interpretations, we model the impact of the LE and HE domains on the exciton transport in this low-temperature regime. This simulation uses a finite element approach to solve the corresponding 2D drift-diffusion equation to describe how the local exciton density *n*(*r⃗*, *t*) evolves in time *t* and space *r⃗*
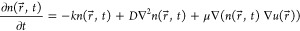
2where the first term on the right-hand side corresponds to exciton recombination (*k* is the recombination rate constant) and the second term to exciton diffusion (*D* is the diffusion coefficient). The third term accounts for the drift of excitons inside the energy gradient, with μ being the exciton mobility and *u*(*r⃗*) being the local energy potential felt by an exciton located at position *r⃗*. We fixed the value of several parameters to our experimental results at 10 K (see SI Section XXI for details): specifically, we use distinct exciton recombination rates extracted from TRPL decays (Figure 5f) for the HE and the LE domains of *k*_HE_ = 0.6 ns^–1^ and *k*_LE_ = 0.01 ns^–1^, with *k* = *k*_HE_ + *k*_LE_. Furthermore, we model the energy landscape of the sample at 10 K as being composed of adjacent domains of both HE (energy range 1.85–1.91 eV) and LE (broader energy range of 1.60–1.85 eV) using the spatial distribution from the PL maps in Figure 4d and the PL spectra in Figure 3a. Thus, *D* is the only adjusted parameter. The finite difference mesh employed to solve the equation consists of a 256 × 256 grid, resulting in individual domain sizes equivalent to 80 nm. Using these conditions, we simulated the spatiotemporal evolution of the excitons at 10 K (Figure 5c,d) and qualitatively reproduced the main features of the dynamics; in particular, the fast spreading of the exciton distribution with spreading coefficient *D* = 0.68 cm^2^ s^–1^ (Figure 5c; see SI Section XXI and Figure S30 for other simulations) closely matches the experimental value of 0.69 cm^2^ s^–1^ (Figure 5e). We highlight here that full quantitative agreement is beyond the scope of this study because it would require a quantitative evaluation of the local concentration and energy of LE states with a nanoscale resolution that cannot be reached with optical microscopy. Nevertheless, the results from this model validate our understanding of the observations in which the local energy gradient drives the excitons to move downward in energy, which results in a lateral movement of excitons from the HE to the LE domains. The fact that LE sites can saturate allows the exploration of further LE sites, leading to long-distance energy transport (up to ∼600 nm) through this downhill energy-transfer effect.

On the basis of our collective results, we propose a general description of the exciton dynamics in these RPP flakes that is summarized in Figure 5a−b;g−i. At room temperature and down to ∼100 K, exciton transport is governed by an interplay between nonradiative trap states and phonons. In this picture (Figure 5a,g), exciton diffusion occurs via a hopping process in which significant thermal energy provided by phonons is required to promote excitons from localized states toward the excited-state continuum. Such a diffusion process is efficient at high temperatures, though this also means that excitons can more readily find deep trap states on which they recombine nonradiatively, hence the PL efficiency generally increases with decreasing temperature. Diffusion becomes less efficient at lower temperature as fewer phonons states are populated (Figure 5h), and at or below 100 K, the exciton–phonon coupling effects freeze out due to a negligible population of optical phonons^11,36,37^ in such RPP flakes. At ∼130 K, a series of broad, lower-energy (LE) states are detectable and are connected to the heterogeneous static disorder in the material. At temperatures below 100 K, excitons are split into two populations, one of which is localized before recombining, leading to the HE resonance. In parallel, a fraction of these excitons moves laterally as they transfer down into the LE sites (Figure 5b,i). Remarkably, this downhill energy-transfer process recovers long-range exciton transport (∼600 nm) at low temperature despite the absence of a phonon population.

We have shown here that the exciton dynamics in these RPP flakes are highly tunable and could be exploited to design tailored optoelectronics devices. For example, one could tune the transport length of excited species over several orders of magnitude by implementing a controlled design of the spatial distribution of the LE states via chemical routes.^50,51^ Furthermore, the saturation of the LE sites means that an external operator could be used to turn their properties on and off at will, for instance, by modulating the exciton density through the illumination intensity or injection rate. This effect introduces the possibility of light-emitting devices in which the dominant emission wavelength can be selectively tuned. Finally, because these LE states are connected to the static disorder and the phononic properties of these flakes, one could design mechanical and/or vibrational^52^ strategies to modulate them in optomechanical device architectures.^52,53^

In conclusion, we studied the local temperature-dependent dynamics and transport of excitons in thin Ruddlesden–Popper perovskite flakes using time-resolved luminescence microscopy. We identified the existence of several temperature regimes and highlighted the interplay between excitons, phonons, and the local energy landscape. Below 100 K, the exciton diffusion process is inefficient due to the absence of optical phonons, yet we showed that excitons can still travel through a static disorder landscape scheme in which long-lived radiative shallow states play an important role. At higher temperatures, the progressive population of the phonon modes causes dynamic disorder to dominate static disorder. In parallel, the exciton–phonon coupling allows the excitons to move across the crystal in a diffusive fashion, hopping from one shallow trap to the next until they either recombine radiatively or they encounter nonradiative deep trap states. Phonons induce dynamic disorder, increasing the exciton mobility and enabling the excitons to reach nonradiative deep traps. This study provides a new microscopic visualization of the fundamental mechanisms underlying the exciton dynamics in these RPP flakes and highlights the major role played by the energy landscape in the diffusion process in such 2D materials. With fine control over such a landscape and, in particular, the presence of nonradiative deep traps states, we could control the efficiency of the excitonic motion and design highly efficient optoelectronic devices based on these Ruddlesden–Popper perovskites. Further work will be required to understand the nature of the low-energy states to allow facile material control over their formation and distributions.

## Supporting Information Available

Details on the sample preparation; the microscopy PL and cryogenics setup; additional photoluminescence characterization of the samples; diffusion measurements at different temperatures; complementary results on *n* = 2 RPP crystals; low-temperature spectral and temporal study of *n* = 4 crystals; and further details of the simulations (PDF)
