# Supplementary material for: Local Energy Landscape Drives Long-Range Exciton Diffusion in Two-Dimensional Halide Perovskite Semiconductors
Source: J Phys Chem Lett. 2021 Apr 20;12(16):4003–11. doi: 10.1021/acs.jpclett.1c00823 (PMC8154849; doi:10.1021/acs.jpclett.1c00823)
Supplement: Supplementary file 1 — jz1c00823_si_001.pdf [file jz1c00823_si_001.pdf]

Local Energy Landscape Drives Long-Range Exciton Diffusion in Two-Dimensional Halide Perovskite Semiconductors

Alan Baldwin [a,b] [§], Géraud Delport[a,] [§,] Kai Leng[c], Rosemonde Chahbazian[a], Krzysztof Galkowski [a,e], Kian Ping Loh[d] and Samuel D. Stranks[a,b]*

a) Cavendish Laboratory, University of Cambridge, JJ Thomson Avenue, Cambridge CB3 0HE, UK. *E-mail: sds65@cam.ac.uk
b) Department of Chemical Engineering & Biotechnology, University of Cambridge, Philippa Fawcett Drive, Cambridge CB3 0AS, UK
c) Department of Applied Physics, The Hong Kong Polytechnic University, Hung Hom, Kowloon, Hong Kong, China.
d) Department of Chemistry, National University of Singapore, Singapore, Singapore.
e) Institute of Physics, Faculty of Physics, Astronomy and Informatics, Nicolaus Copernicus University, 5th Grudziadzka St., 87−100 Toruń, Poland

[§] These authors contributed equally.

I. Synthesis of the crystals and samples preparation:

Synthesis of $(C_4H_9NH_3)_2(CH_3NH_3)_3Pb_4I_{13}$ ($BA_2MA_3Pb_4I_{13}$; n = 4) bulk single crystal. A temperature-programmed crystallization method was applied to synthesize n = 4 RPP single crystal[17]. PbO (0.69 M), BAI (0.17 M) and MAI (0.52 M) precursors were dispersed in a concentrated HI and H_3PO_2 mixture (7.6:1, vol/vol) in an Ar-filled glove box, and then heated at 110 °C with stirring for 40 min to give a clear yellow solution. The solution was quickly transferred to an oven at 110 °C and allowed to cool slowly to room temperature at a rate of 3 °C h^{-1}, where upon metallic black square- or rectangle-shaped crystals started to form. The crystals were isolated by vacuum filtration and dried in an Ar-filled vacuum chamber at room temperature.

Synthesis of $(C_4H_9NH_3)_2(CH_3NH_3)_3Pb_4I_{13}$ ($BA_2MAPb_2I_7$; n = 2) bulk single crystal. PbO (0.59 M), BAI (0.43 M) and MAI (0.31 M) precursors were dissolved in a concentrated HI and H_3PO_2 mixture (9:1, vol/vol) in an Ar filled glove box. The subsequent steps are same as the preparation of n = 4 described above.

RPP exfoliation and protection. Thin RPP flakes are produced by mechanical exfoliation of their bulk single crystals. Scotch tape is applied to bulk crystal surface before adhering to a SiO_2 substrate. Following this the flakes are encapsulated with a hexagonal boron nitride (hBN) layer. All the photoluminescence studies described herein were carried out with the exfoliated perovskite flake protected by the hBN.

II. Details of the photoluminescence microscopy setup:

The time-resolved photoluminescence (TRPL) images and diffusion measurements were measured using a confocal microscope setup (PicoQuant, MicroTime 200, see Figure S1) The excitation laser, a 510-nm pulsed diode (PDL 828, PicoQuant, pulse width of around 100 ps), was directly focused onto the sample with an air objective. The emission signal was separated from the excitation light using a dichroic mirror. A pinhole of 50 μm was included in the detection path, as well as an additional 510-nm longpass filter to minimise the laser contribution to the recorded signal. The TRPL was then focused onto a Hybrid PMT detector connected to a PicoQuant acquisition card for time correlated single-photon counting (time resolution of 100 ps). Repetition rates of 10 MHz were used for the maps and the diffusion profiles, depending on the configuration.

III. Installation of a cryostat for the temperature dependent measurements:

For the TRPL and diffusion mapping as a function of temperature, the perovskite crystals were placed under vacuum and mounted to the cold finger of a constant-flow liquid helium cryostat (Oxford, MicroHires).

IV. Confocal and Diffusion mapping configurations:

The raster scanning was performed using a Galvano mirror system while both the objective and the sample remain at a fixed position. In the case of conventional local TRPL measurements with this setup, both the excitation and the emission are scanned through the mirror system. For diffusion mapping, the emission was scanned using the Galvano scanner, with the excitation source reaching the sample through an alternate path avoiding the scanners

allowing the excitation position remain fixed (x = 0), to create the diffusion profiles from the main text.

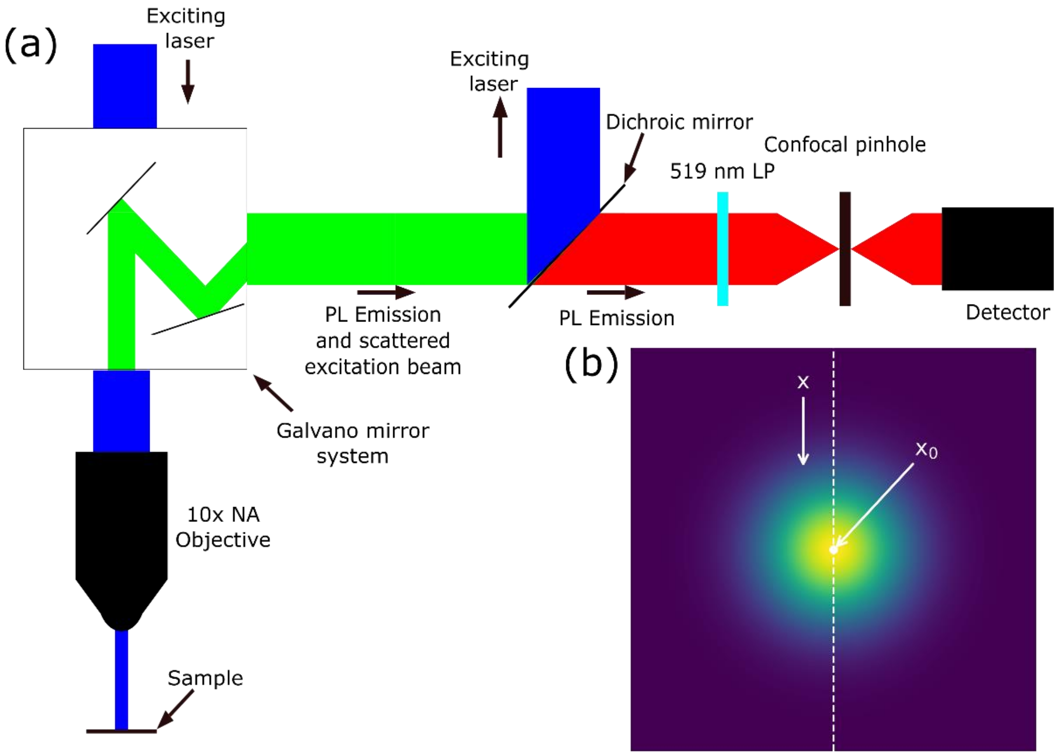

Figure S1: Diffusion measurement technique. (a) Confocal microscope configuration for diffusion measurements. Excitation laser show in blue, PL emission and scattered laser in green, PL after scattered laser removed in red. In contrast to the normal working mode the excitation source does not enter the galvano mirror system. (b) The sample excitation is fixed at x = 0, x_0, while the PL emission, heatmap, is scanned along the x axis, dashed white line.

V. Free carriers

The Saha equation[4], also called the 2D mass action law allows us to evaluate the proportion of photogenerated the free carrier fraction (and excitons) as a function of the total carrier density, based on the hypothesis that excitons and free carriers are in quasi-equilibrium.

$$\frac{x^2}{1-x} = \frac{1}{n}\left(\frac{2\pi\mu k_b T}{h^2}\right) e^{-\frac{E_b}{k_B T}}$$

In which x is the fraction of free charge, $\frac{n_{fc}}{n}$, and n is the total density of excitation $n = n_{fc} + n_x$. μ is the reduced mass of the exciton (approximated to 0.20 m_e)[5], T the temperature and E_b the exciton binding energy. The values of the exciton binding energies E_b are respectively 140 and 240 meV for n=4 and n=2 members of this RPP series[5].

Note that for the data presented for the lowest fluence room temperature measurements free carriers are dominant.

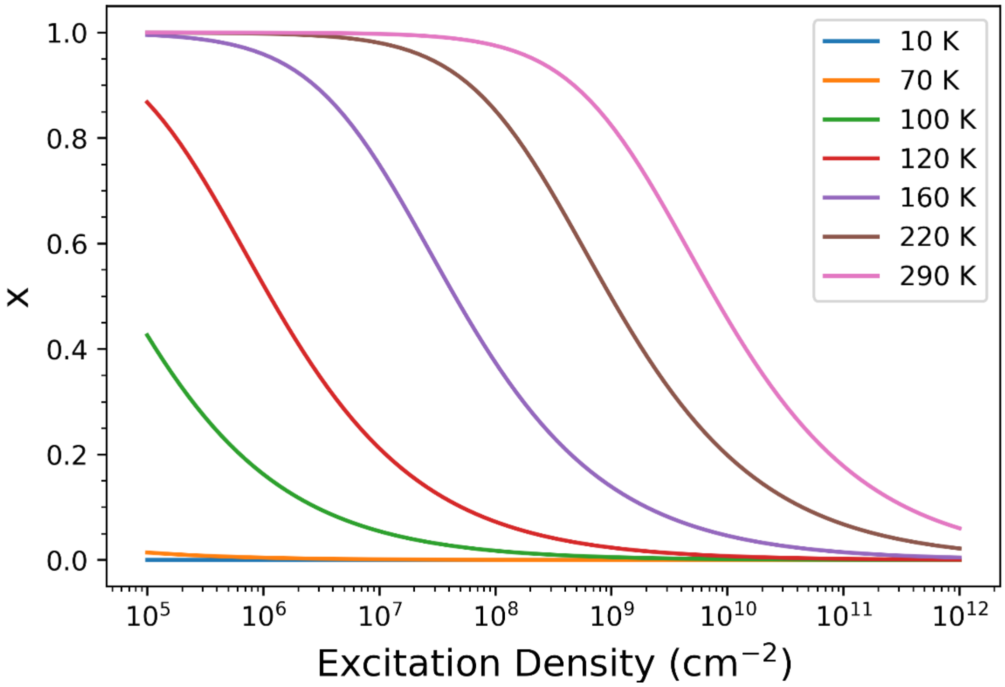

Figure S2: Free charges fractions. Calculated according to the Saha equation as function of the density for the n = 4 sample, shown for different temperatures relevant to this study.

VI. Transport length scales:

The transport length can be calculated as the difference, Δσ, between the standard deviation, σ_f, of the PL signal at the end of the measurement (t=∞) and that at the start of the measurement, σ_s, (t=0). This will give an upper bound on the transport lengths that can be achieved. Here we use the data from measurements at 10 K to calculate this upper bond. From the data in Figure 4a in the main text at later times, 70 – 80 ns, it is clear the transport has ceased. The

mean of the last 10 data points in this graph provides σ_f^2 = 1.599 $\pm$ 0.004 μm^2. Taking this from the original σ_i^2= 1.25 $\pm$ 0.05 μm^2 yields a transport length of 591 $\pm$ 12 nm. Another approach, is to evaluate the statistical diffusion length L_d, would be to use the formula:

$$L_d = \sqrt{\frac{D}{\tau}}$$

in which D is the exciton diffusion coefficient and τ is the effective lifetime of the excitons extracted from the TRPL decays curves. This approach generally yields a smaller transport length value than the maximal transport length described above, but it may be more representative of the mean behaviour relative to the global exciton population

VII. Determination of the number of RPP layers via optical contrast imaging:

We applied the optical contrast imaging method[1] (see Figure S3) to evaluate the thickness of the flake to be ≈ 9 stacked quantum wells (≈30 nm thickness). The smallest observed variation of the contrast ~10 arb.unit, is likely to correspond to the addition (or suppression) of a monolayer. Therefore, the thickness of flake of interest is estimated to be of 9 monolayers (~ 30 nm), given that its optical contrast is of ~ 90. Despite the fact that our optical characterization method is not fully quantitative (ie without the use of the AFM that was used in our parent publication on which our study is based), we estimate that the thickness estimation error is small enough not compromise our hyptotheses on the fact that the photon recycling and the phase transition process are made unifficient in such thin RPP crystals.

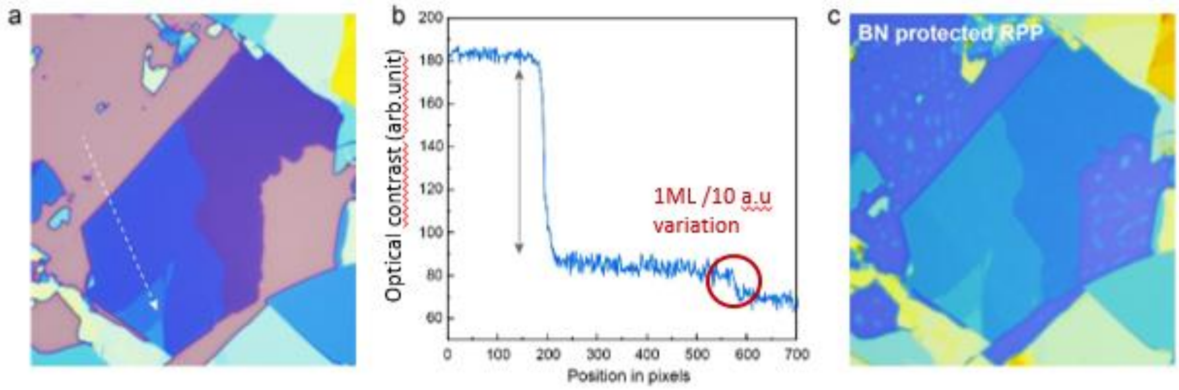

Figure S3: Determination of RPP layers by optical contrast imaging. (a) Optical image of n = 4 RPP flakes exfoliated on SiO_2/Si substrate obtained in a reflection configuration. The different parts of the flakes exhibit different optical contrast (and colours). The optical contrast is a direct signature of the number of stacked quantum wells at a particular location of the sample. **(b)** Evolution of the optical contrast obtained along cross-section line on (a). (c) Optical image of BN protected *n* = 4 RPP flakes with the same area in (a).

VIII. Phase Purity:

Figure S4 shows high-resolution X-ray diffraction patterns of our synthetic single crystals $BA_2MA_{n-1}Pb_nI_{3n+1}$ (n = 1, 2, 3, 4). The XRD data testifies to the phase purity of our RPPs (n = 1, 2, 3, 4) in terms of the excellent agreement of the peak position and number of the low-angle peaks compared with other works[1,2].

The thin single crystal RPPs shown in our manuscript is exfoliated from bulk single crystals. As shown in Figure S4b, from bulk to molecularly thin, the full width at half maximum (FWHM) of the PL has decreased with thickness indicating that exciton-phonon coupling is reduced for thinner flakes, or that there is negligible increase in the density of defects in thin crystals compared with bulk ones[3].

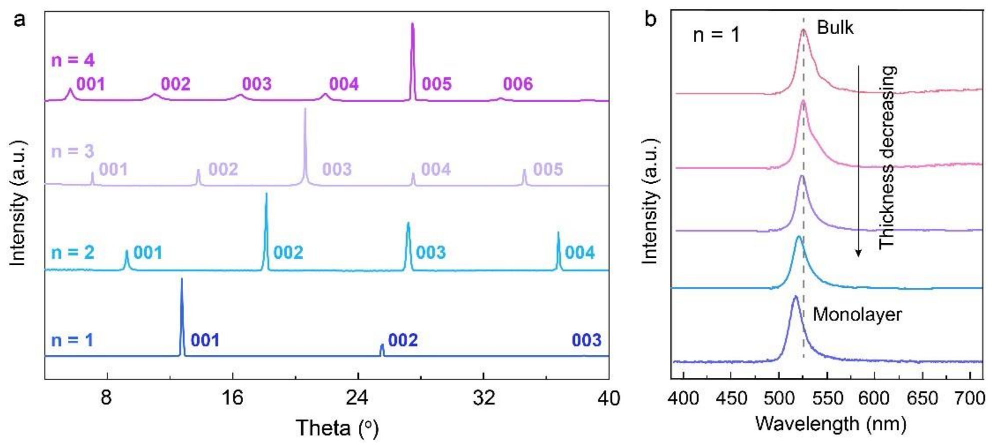

Figure S4: High-resolution X-ray diffraction patterns of $BA_2MA_{n-1}Pb_nI_{3n+1}$ (n = 1, 2, 3, 4) single crystals. Phase purity is established by monitoring the position and number of the low-angle peaks in X-ray diffraction patterns for each *n* value. The diffraction patterns only show (00L) orientation further indicate a highly ordered layered stacking arrangement and high quality of single crystals for all samples. (b) The PL emission of BA_2PbI_4 as its thickness decreases from bulk to monolayer.

IX. PL spectra measurements and hyperspectral mapping of the perovskite flake of interest:

Unless mentioned otherwise; all the PL spectra included in this paper were measured directly inside our confocal PL microscope (including the temperature dependent PL spectra) with a 510 nm laser. To obtain these spectra, the collection signal of the microscope was diverted to ANDOR Kymera 193i spectrograph with a 600 lines per mm blazed at 500 nm, coupled to a CCD Array camera.

The only exceptions are the PL spectral maps of Figure S5 and the spectrum in Figure S6, which were performed with a wide-field microscope (IMA VISTM, Photon Etc.). We employed a wide field air objective of 20x magnification (0.45 Numerical aperture (NA)) to characterise the flake in reflectance mode. The PL measurements were carried out using

excitation from a 405 nm continuous wave laser, focused on the sample surface. The reflected laser beam was removed through use of a 420 nm long pass filter allowing collection of the emission spectrum of the sample. To obtain spectrally resolved images, a volume tuneable Bragg filter was employed.

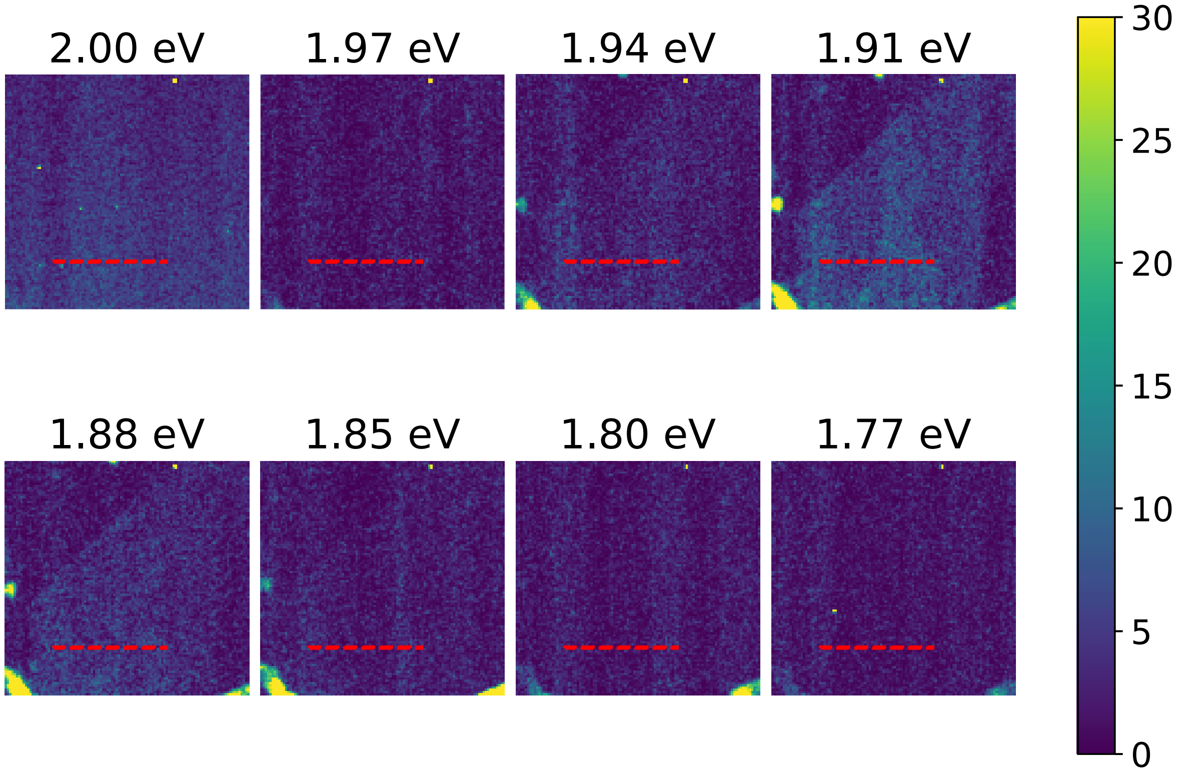

Figure S5: Room temperature hyperspectral maps for the n = 4 flake, with the investigated area shown in red. To increase the contrast the 2 eV image, showing only noise, was used as an estimate of the background, with all other images subtracted having this subtracted having this subtracted. The 2 eV image is shown as recorded less 285 counts to ensure the common colour bar remains applicable for all images.

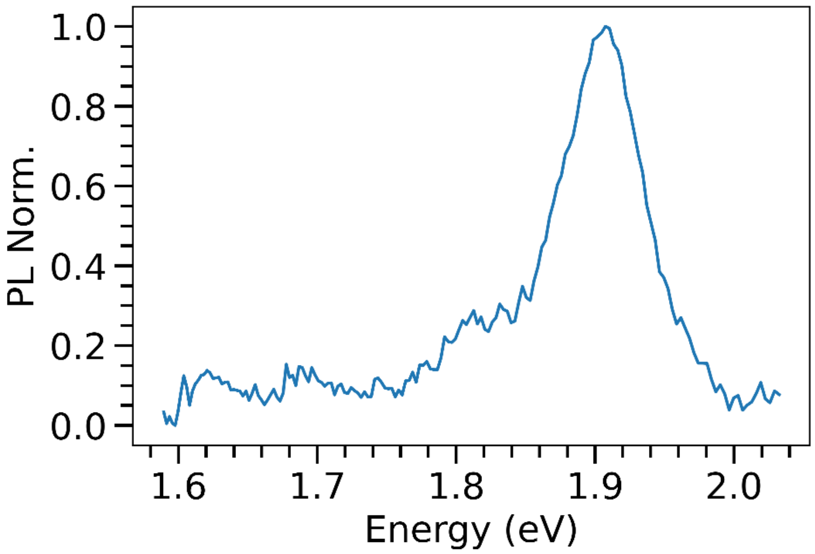

Figure S6: Spatially integrated PL spectrum of the investigated n = 4 flake, obtained from hyperspectral mapping, section VIII , normalised for clarity. With a resonance around 1.9 eV, this spectrum corresponds to previously reported values[6] for n=4 crystals.

X. Additional PL data for the excitation density series at room temperature:

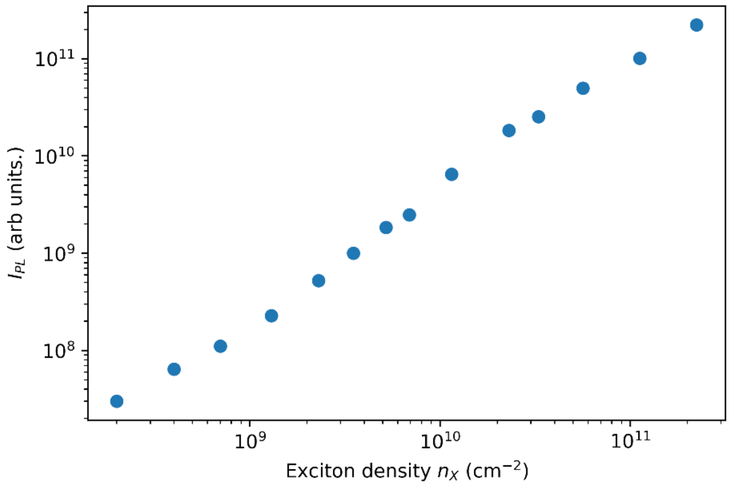

Figure S7: Evolution of the PL intensity as a function of exciton density of the n=4 RPP flake, from which the values of the parameter η displayed in Figure 1d are calculated using the formula $\eta = I_{PL}/n_X$. The superlinear evolution of the PL intensity with fluence, that occurs for excitons densities between 1 x 10^9 and 2 x 10^{10} cm^{-2} indicates that the light induced trap filling process is taking place.

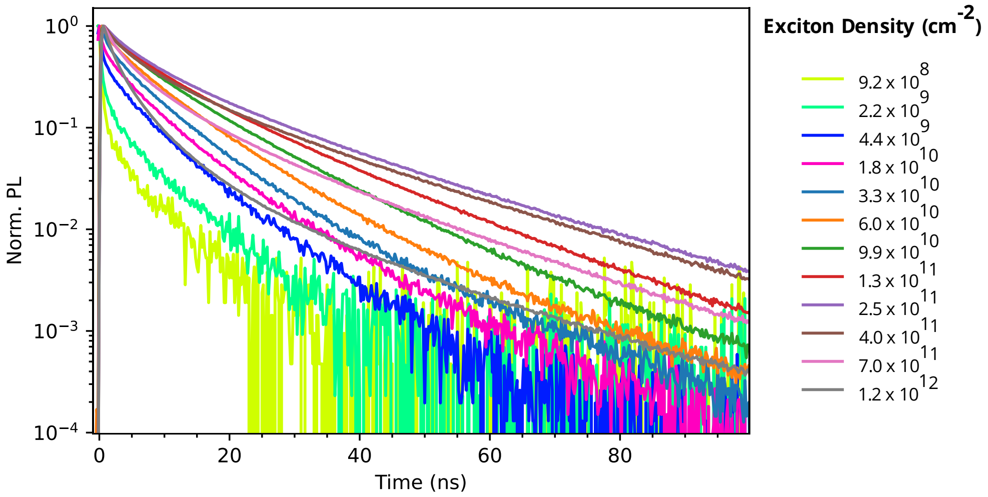

Figure S8: Normalized TRPL decays of the n = 4 flake, featuring more excitation densities than displayed in Figure 1c.

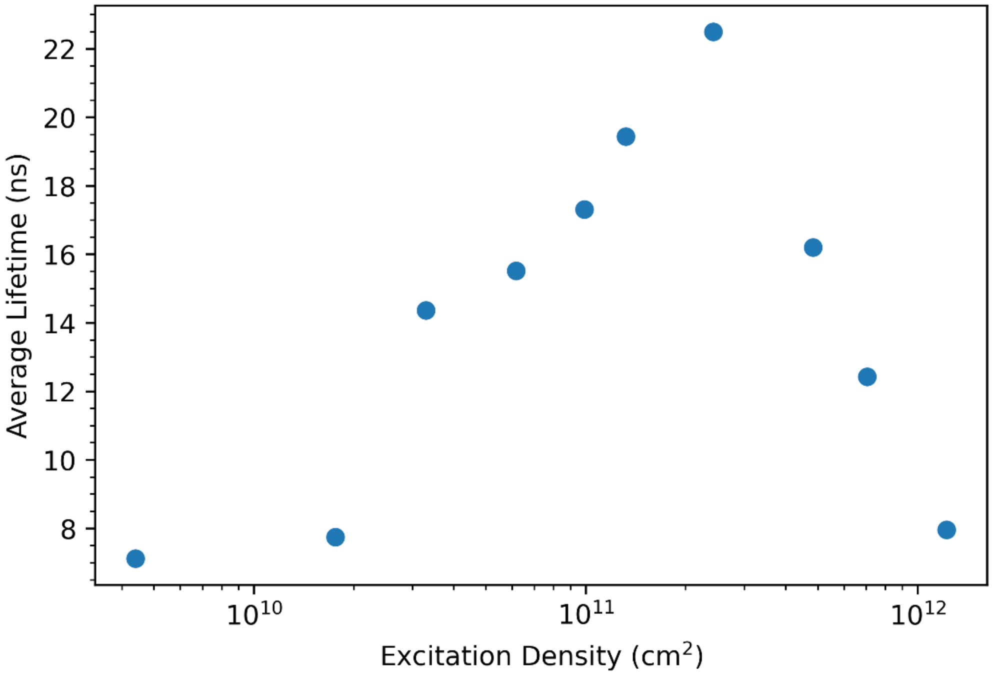

Figure S9: 1/e lifetime of the n=4 flake at room temperature as a function of excitation density. This is an extended version of the inset in Figure 1(e).

XI. Used objective lenses and optical resolution:

For the PL maps (Figure 1c and 2a) and the diffusion measurements displayed in (Figure 2b), a 100x air objective of 0.8 Numerical aperture (NA) was used. In this configuration, the lateral

spatial resolution is of the order ~500 nm. As a demonstration of this, the standard deviation of the measured Gaussian beam at t = 0 in Figure 2a is of σ(0) = 528 nm

For the rest of the diffusion measurements and TRPL mentioned in the main text (Figure 2c, 3 and 4), a 10x objective lens with a 0.4 NA has been used. Indeed, we found such a low magnification objective has a long working distance that is more suitable for the cryogenic measurements. As seen in Figure S10, this objective lens generates excitons over a larger spatial profile (σ(0) = 2165 nm), but does not alter the diffusion measurements.

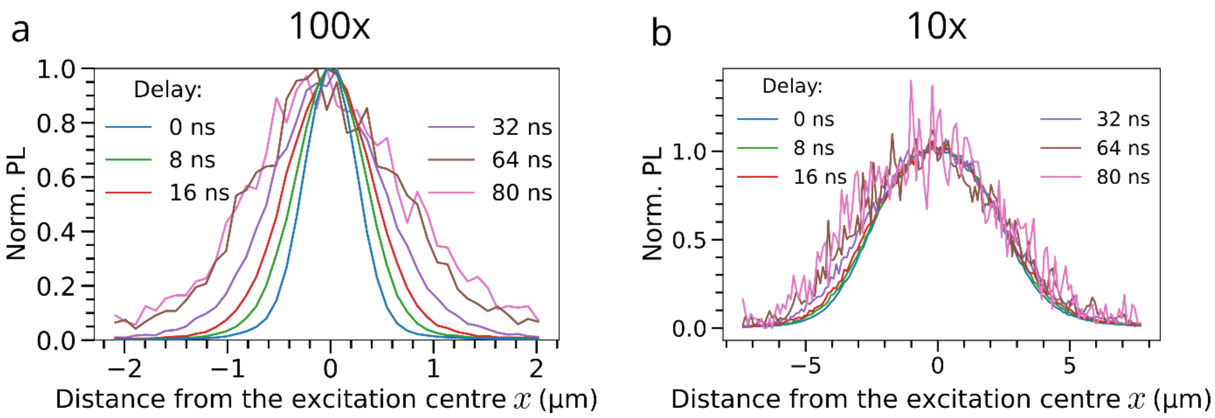

Figure S10: Comparison of PL profiles for the objective lenses used. (a) 100x 0.8 NA objective and (b) 10x 0.4 NA objective. (a) is the same as presented in Figure 2(b). Excitation was via 510 nm 10 MHz laser.

The initial value of σ at t = 0 originates from a combination of factors, including the optical resolution of the setup and the possibility of early time diffusion or reabsorbed photons emitted at early times within the temporal instrument response of the setup (≃385 ps)

XII. Additional Diffusion data for the fluence series at room temperature

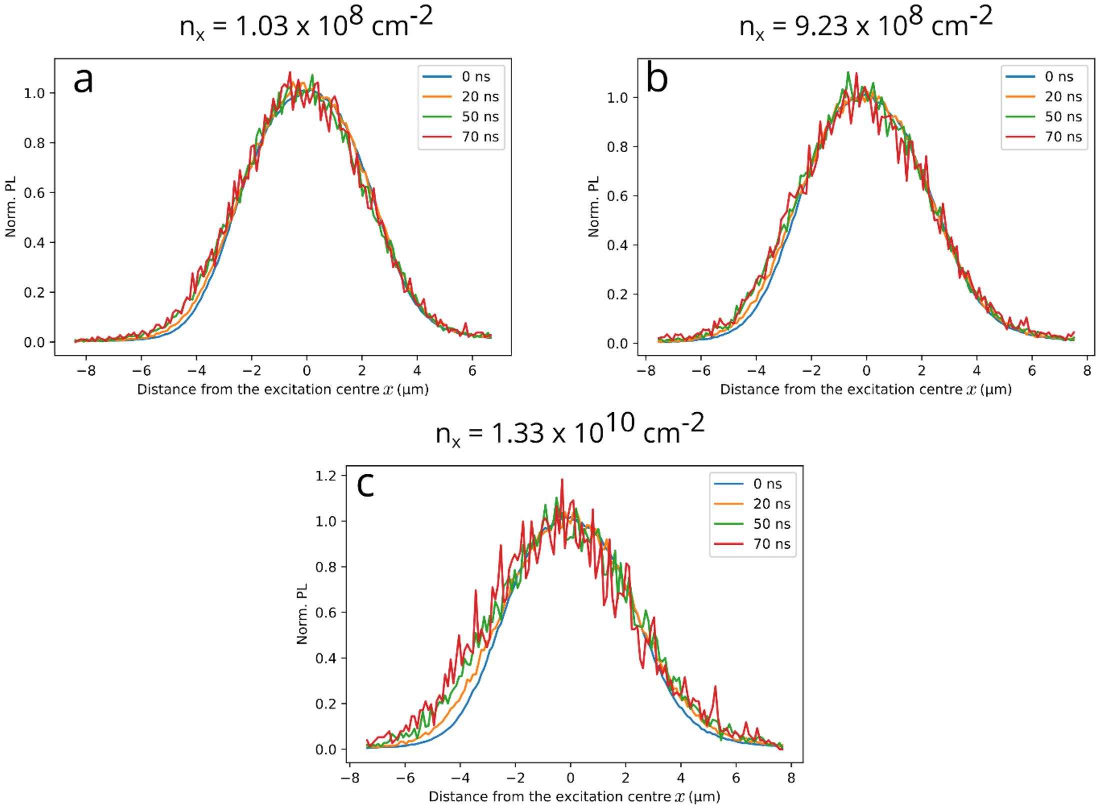

Figure S11: Selected spatial PL profiles, normalised, at different times after excitation by the laser pulse, localised at x = 0 (centre of the red line in Figure 2a) from which the data in Figure 2c is obtained, taken with a 0.4 NA 10x objective lens. While the differences of the lateral width as a function of time seem small on these graphs, they are clearly quantifiable once we apply our Gaussian fitting process. The corresponding excitons densities n_x are mentioned above each graph. The spreading of the spatial profiles becomes more pronounced as $n_{x,}$ as displayed in Figure 2c. Excitation was via a 510 nm laser with a repetition rate of 10 MHz.

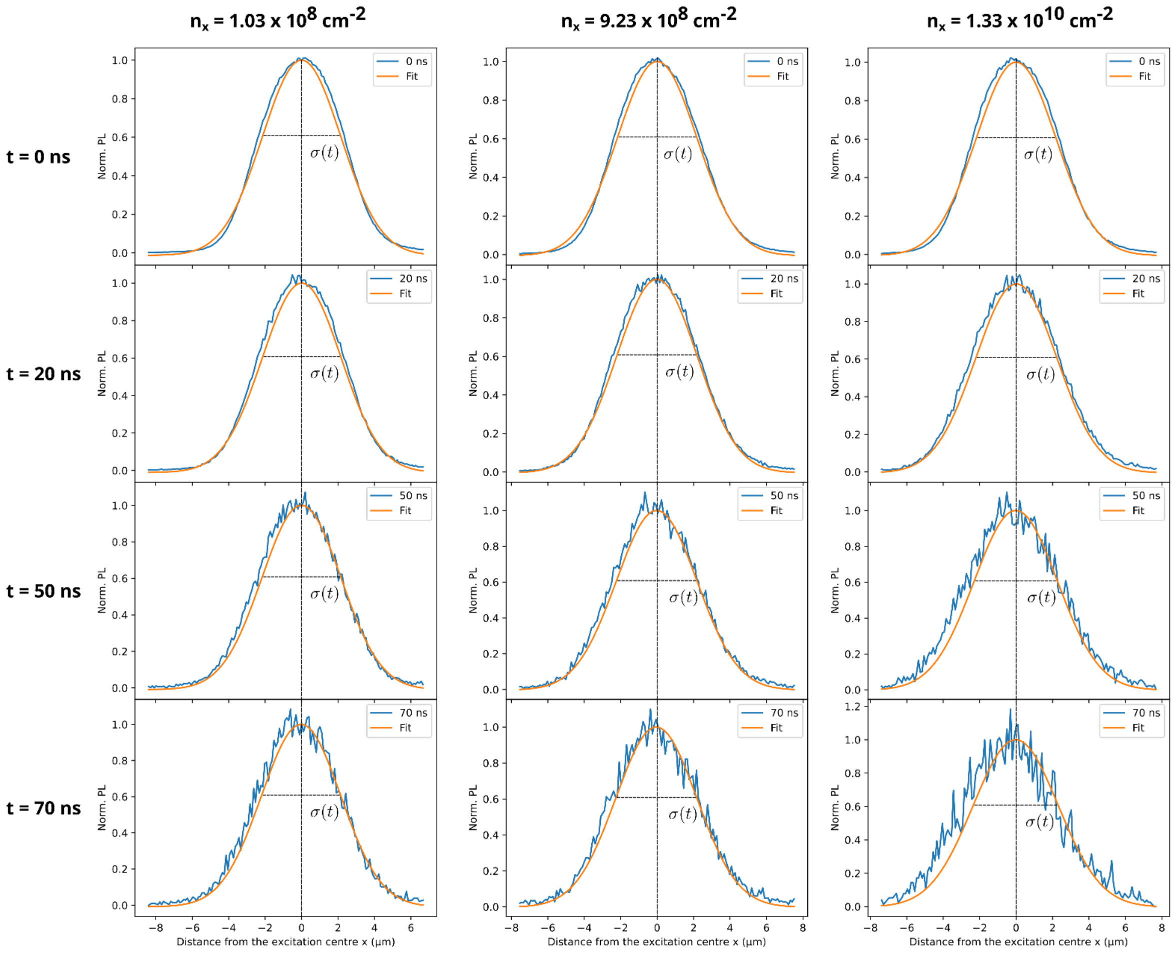

Figure S12: Example Gaussian fits. Gaussian fits of the PL profiles presented in Figure S11. Shown for each of the three fluences and each of the four displayed times. Horizontal dashed lines indicate the standard deviation of the fits. Vertical dashed lines indicate the centre of the PL profile, i.e. x=0.

XIII. Complementary measurements for the n = 2 flake:

These results are displayed to highlight the similarities between the excitonic behaviour within the *n*=4 flakes (in the main text) and the *n*=2 ones (below), despite the different in the size of the quantum wells.

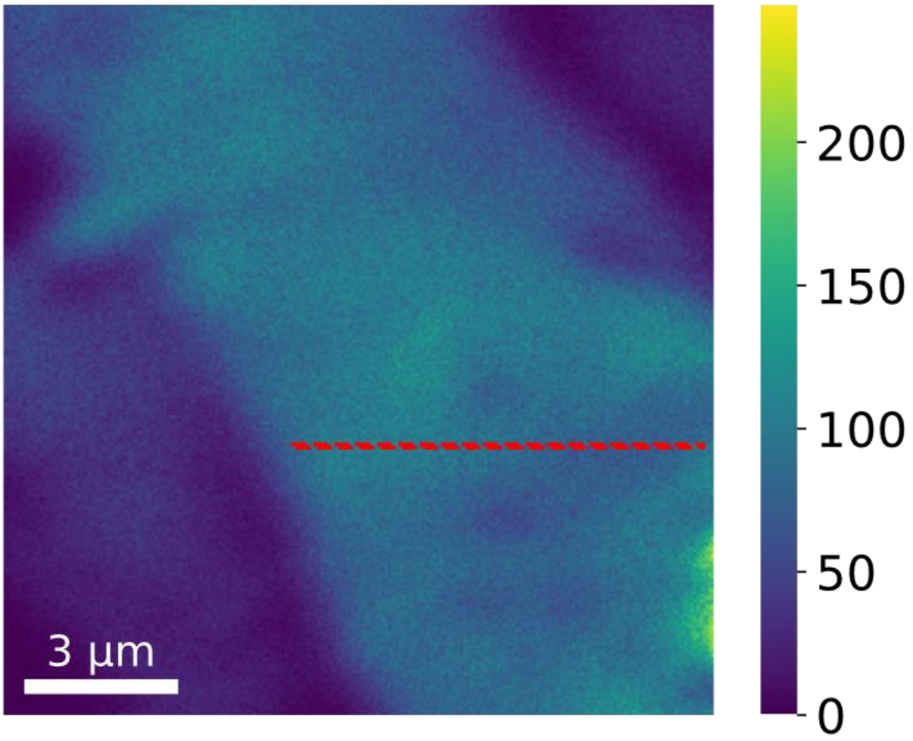

Figure S13: PL intensity map of n = 2 flake (excitation at 510 nm) at room temperature. images. The red strip highlights the region of interest over which transport is subsequently analysed, with the spatial parameter x defined spanning from the centre of the region.

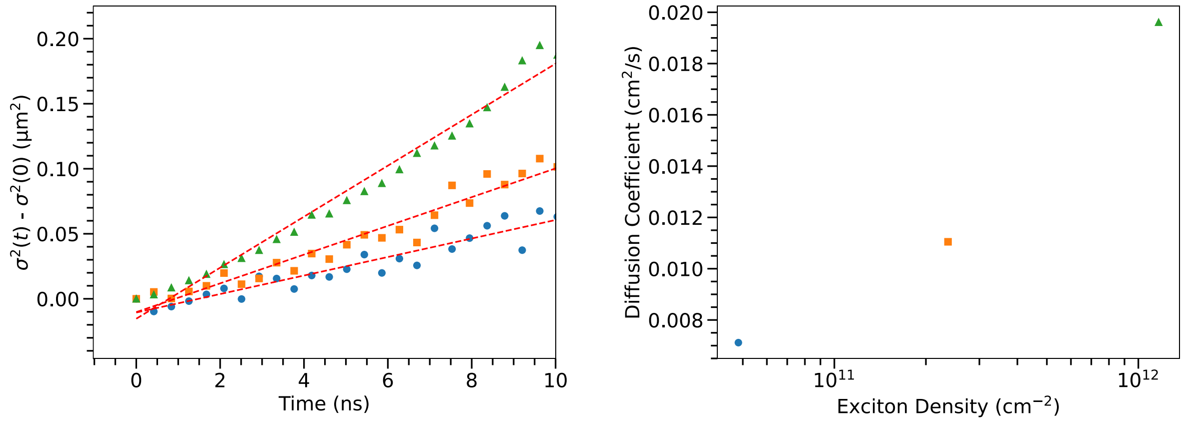

Figure S14: Room temperature fluence measurements for n = 2 flake. (a) Spreading profiles showing the temporal evolution of the squared broadening quantities $\sigma^2(t) - \sigma^2(0)$ of the spatial profiles for the flake at room temperature on the region of interest at three different densities of injected excitons. Fits to Equation 1 are shown in red. (b) Diffusion coefficients extracted from fits to the data with Equation 1 of the main text. As for the n = 4, we see here that the measured diffusion coefficient increases with fluence as the trap filling process take places. This indicates that the presence of traps has an important influence on the excitons motion.

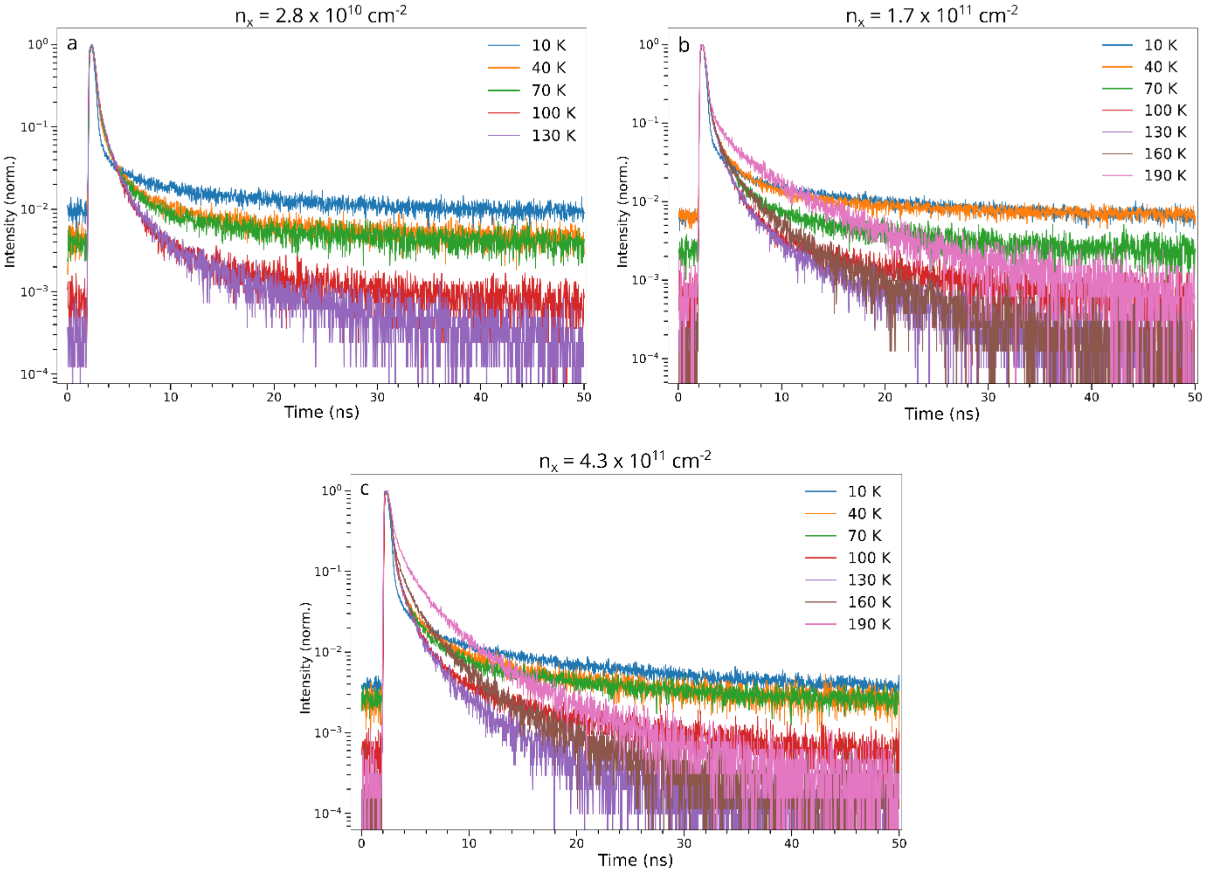

Figure S15: Time-resolved PL decays for the n = 2 flake shown in Figure S13 at different temperatures and excitation densities. Globally showing that the lifetime at early time decreases as the temperature decreases, similarly to the n = 4 flakes.

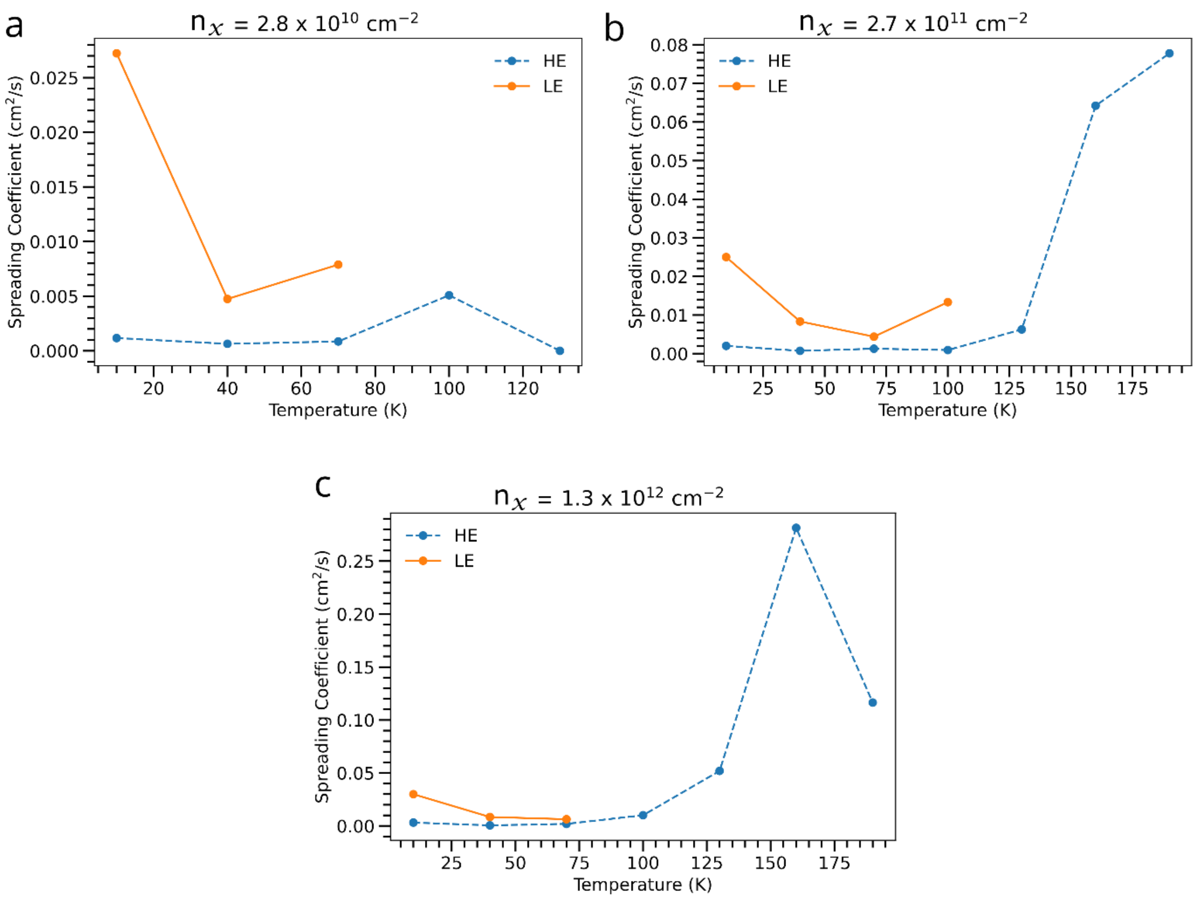

Figure S16: Spreading coefficients extracted from the area of interest of the n = 2 flake from Figure S13. The data has been extracted at different excitation densities, as indicated in the figure. The data above 75 K have been extracted from the spreading curves without the use of bandpass filters. The data below 75 K have not been resolved using bandpass filters, instead following on from our results obtained from the n = 4 flake the LE coefficients have been assigned to the initial fast spreading with the HE assigned to the later time slower spreading.

XIV. Additional PL data for n = 4 at low temperatures:

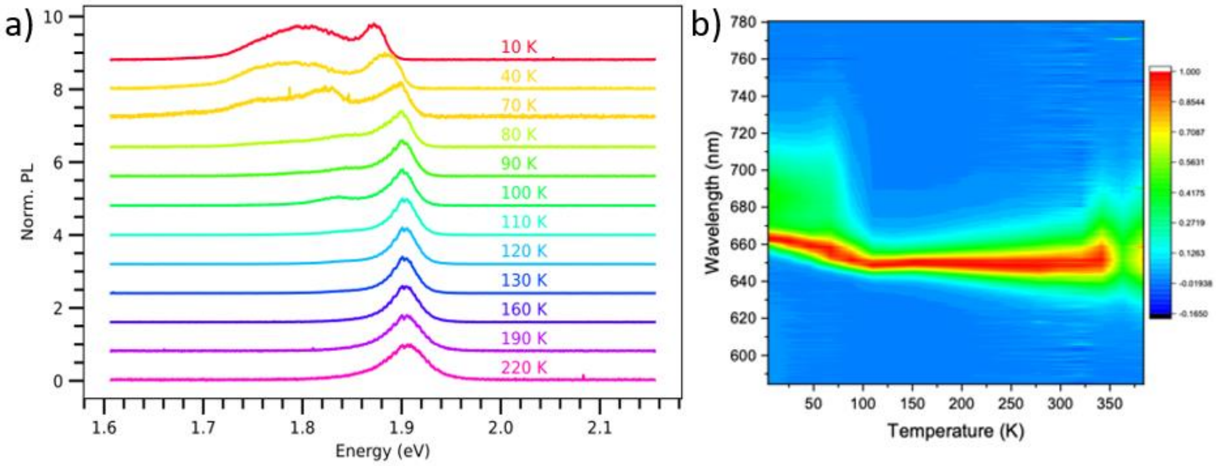

Figure S17: Extended version of Figure 3a, showing the Temperature dependent evolution of the high energy (HE) PL peak and the emergence of the low energy (LE) peak present in the n = 4 flake, with excitation at 510 nm and a repetition rate of 10 MHz and excitation density of 9.2 × 10^8 cm^{-2}. In (a), the different spectra are normalised to the maximum value and vertically offset for clarity. In (b), the spectra have been artificially collated into a contour plot, in which each colour corresponds to a different (normalised) PL intensity. Such contour plot is particularly suitable to highlight the threshold temperature of ~ 130 - 100 K under which the spectral position of the PL maximum starts changing.

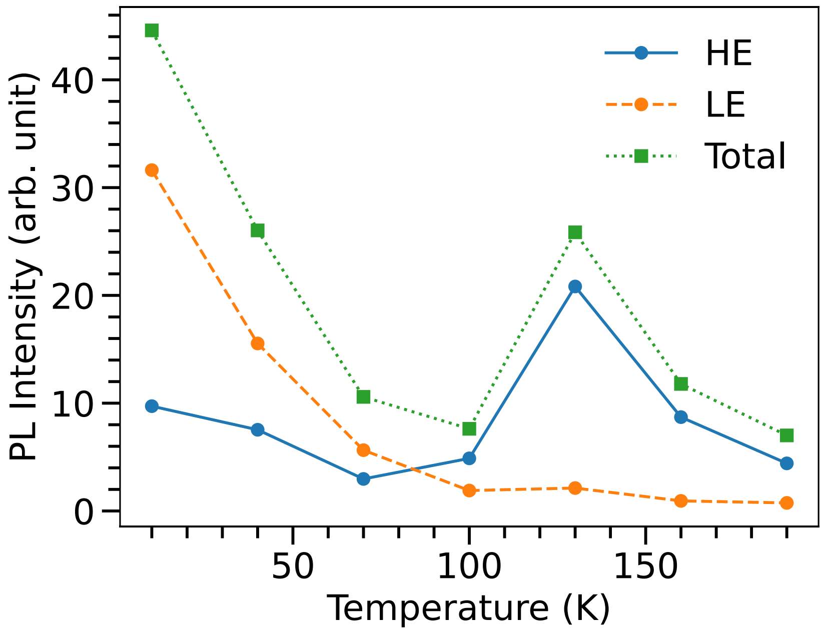

Figure S18: Integrated PL intensity spectrally separated into the low energy (LE) and high energy (HE) components, shown with the total PL signal integral in green. Obtained from the PL spectra in Figure S17. The Intensity is calculated as the integral of the PL counts

over the range of (1.72 - 1.855) eV for the LE region and (1.855 - 1.92) eV for the HE region. The total is the integral of the entire data.

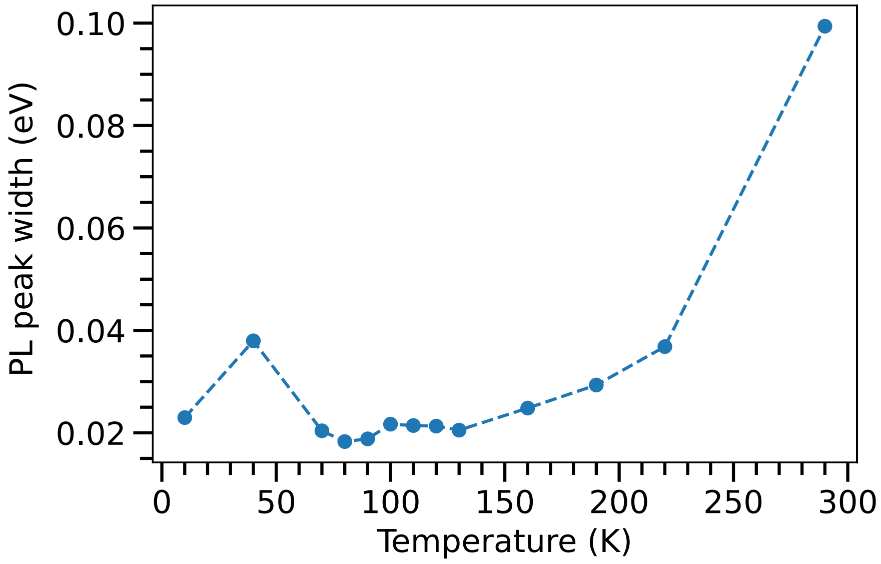

Figure S19: FWHM of the HE resonance in the PL spectra of the *n = 4* flake as a function of temperature, corresponding to the spectra featured in Figures 3a and S17. The broadening the PL peak above 130 K is due the coupling of the excitons to the optical phonons that become available at these temparatures. Obtained by fitting the PL spectra in Figure S17 with Gaussian functions, extracting the standard deviations and converting this to a FWHM via FWHM = $2\sigma\sqrt{2\ln 2}$.

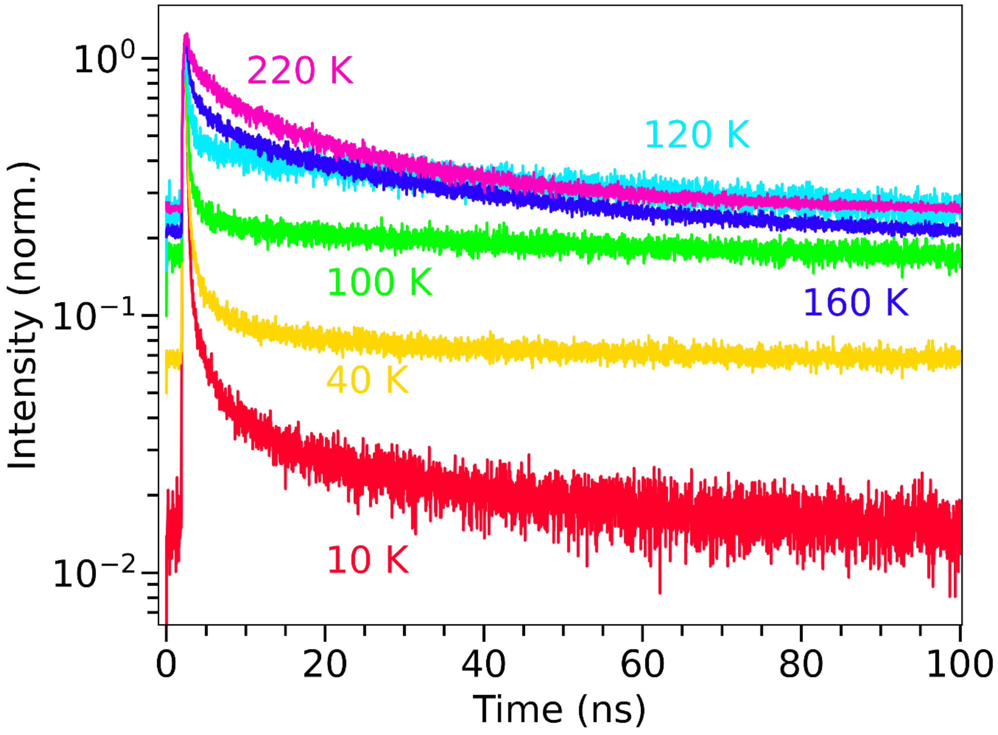

Figure S20: TRPL decays of the n = 4 flake at different temperatures, obtained from the centre of the region shown in Figure 1c. As the temperature decreases, we observed a decrease of the slope at early time (< 5ns) of the TRPL decay curves, associated with the decrease of the 1/e lifetime displayed in Figure 3b. In addition, we see the emergence of a long decay component as the temperature decrease, which is discussed in the main text. Excitation via a 510 nm pulsed laser at 10 MHz excitation density of 9.2×10^{8} cm^{-2}.

XV. Investigation of the effect of excitation density on the LE states:

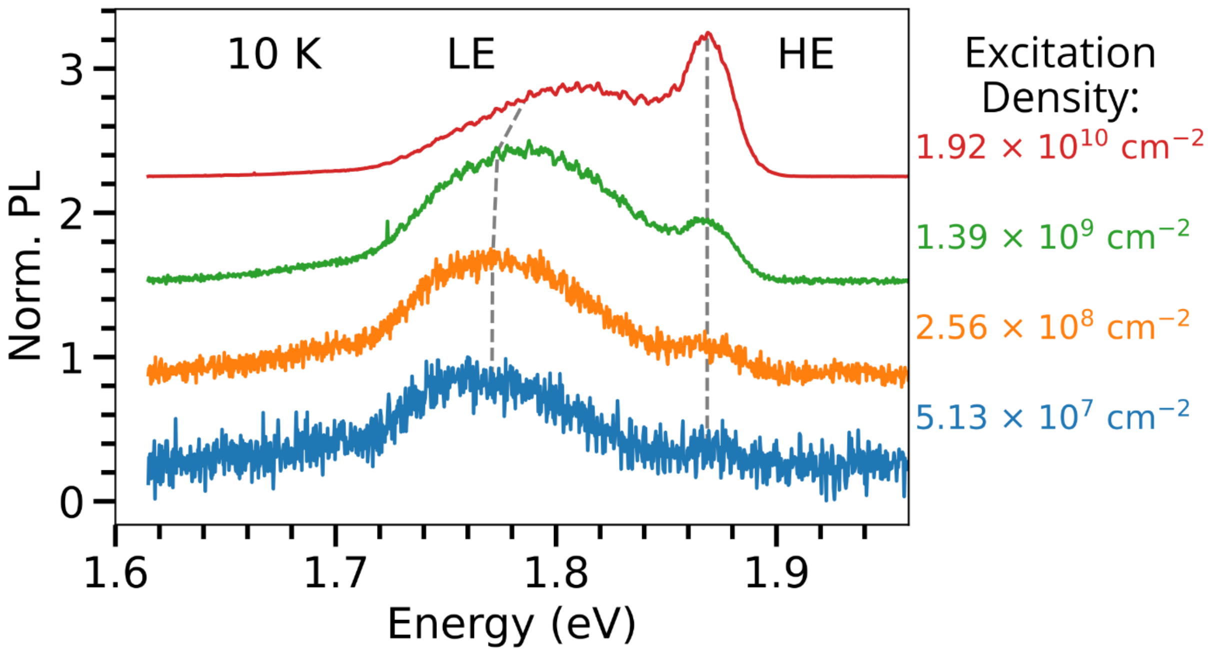

Figure S21: PL spectra of the centre of the region of interest of the n=4 flake obtained at 10 K at difference excitation densities. Saturation of the LE component with increasing excitation density can be seen to occur. Excitation was via a 510 nm laser at a repetition rate of 10 MHz.

XVI. Bandpass filter measurements:

To selectively probe the HE and LE resonances two complementary bandpass (BP) filters, 650 nm (HE) and 700 nm (LE) of 40 nm width, were used. Figure S22 shows the local PL spectra

from the n = 4 flake at low temperatures with the transparency windows of the BP filters indicated.

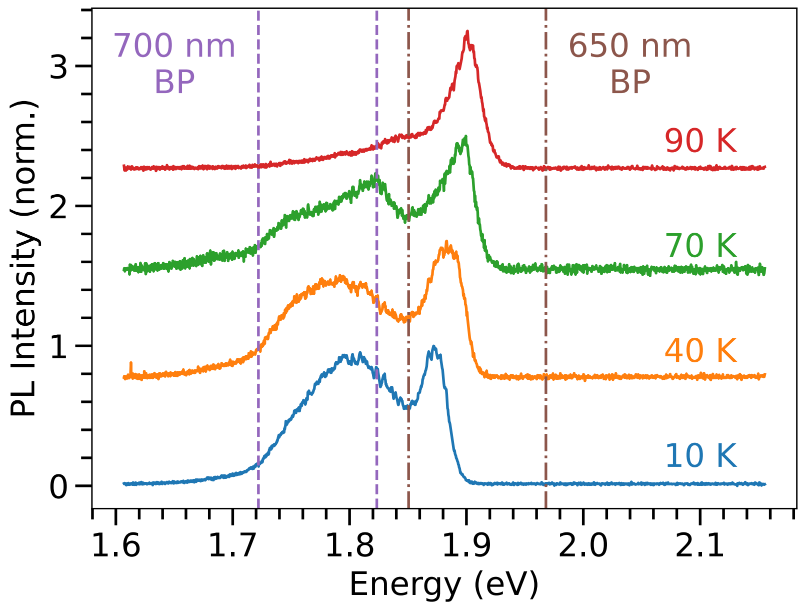

Figure S22: PL spectra at the temperatures band pass filters were used, the limits of the transparency windows of the two band pass filter ranges are indicated by vertical purple and brown lines.

XVII. Band Pass Resolved TRPL Decays at Low Temperatures:

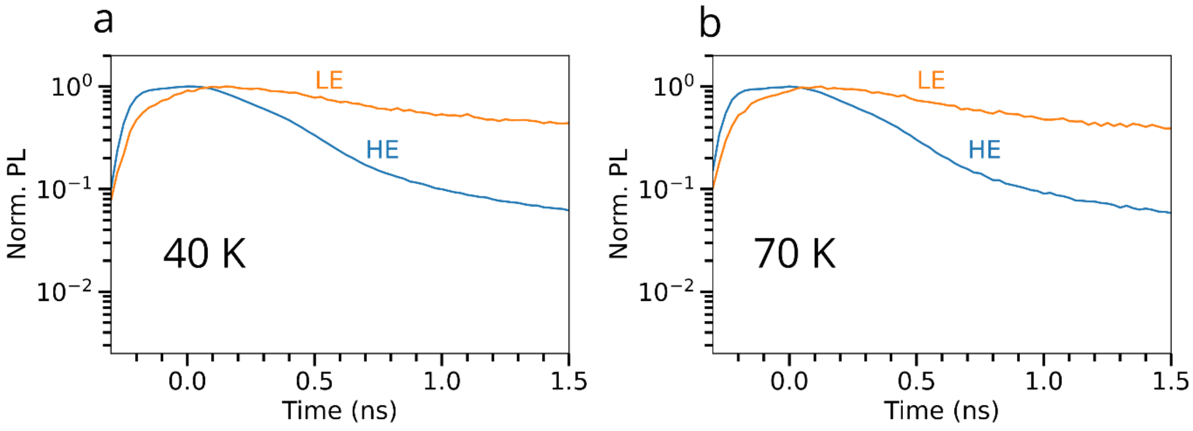

Figure S23: BP resolved TRPL decays at (a) 40 K and (b) 70 K obtained from the centre of the studied region of the n = 4 flake, highlighting the transfer from the rise time of the LE

regions compared to the HE regions. Excitation was via a 510 nm laser at a repetition rate of 10 MHz and an excitation density of 9.2×10^8 cm^{-2}.

XVIII. System IRF:

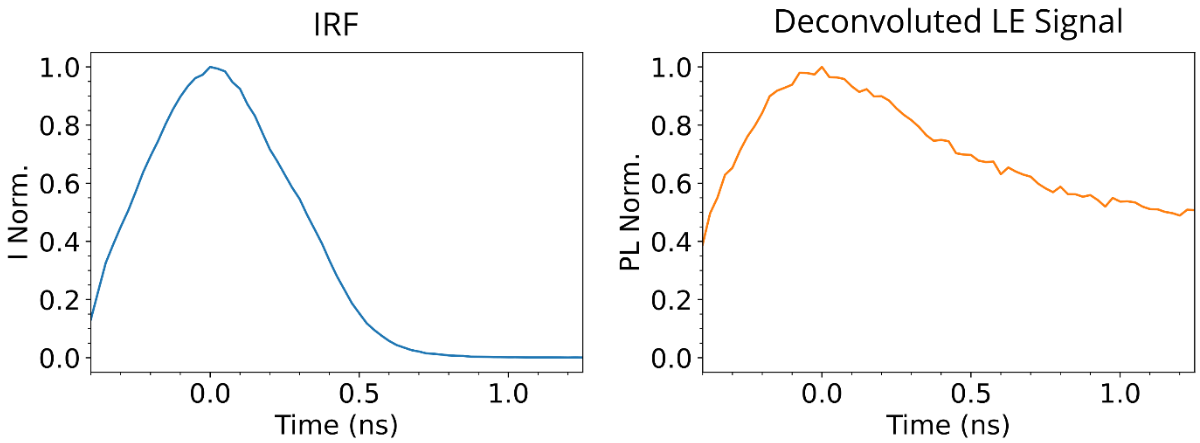

Figure S24: IRF data from the setup. (a) IRF of the setup taken by shining a 510 nm laser directly on the detector. (b) LE TRPL decay, from Figure 3c, after deconvolution from the IRF in (a).

XIX. Additional Diffusion profiles and extracted data:

$$\sigma^2(t) = \sigma^2(0) + 2Dt \qquad (1)$$

Equation 1 reproduced from the main text.

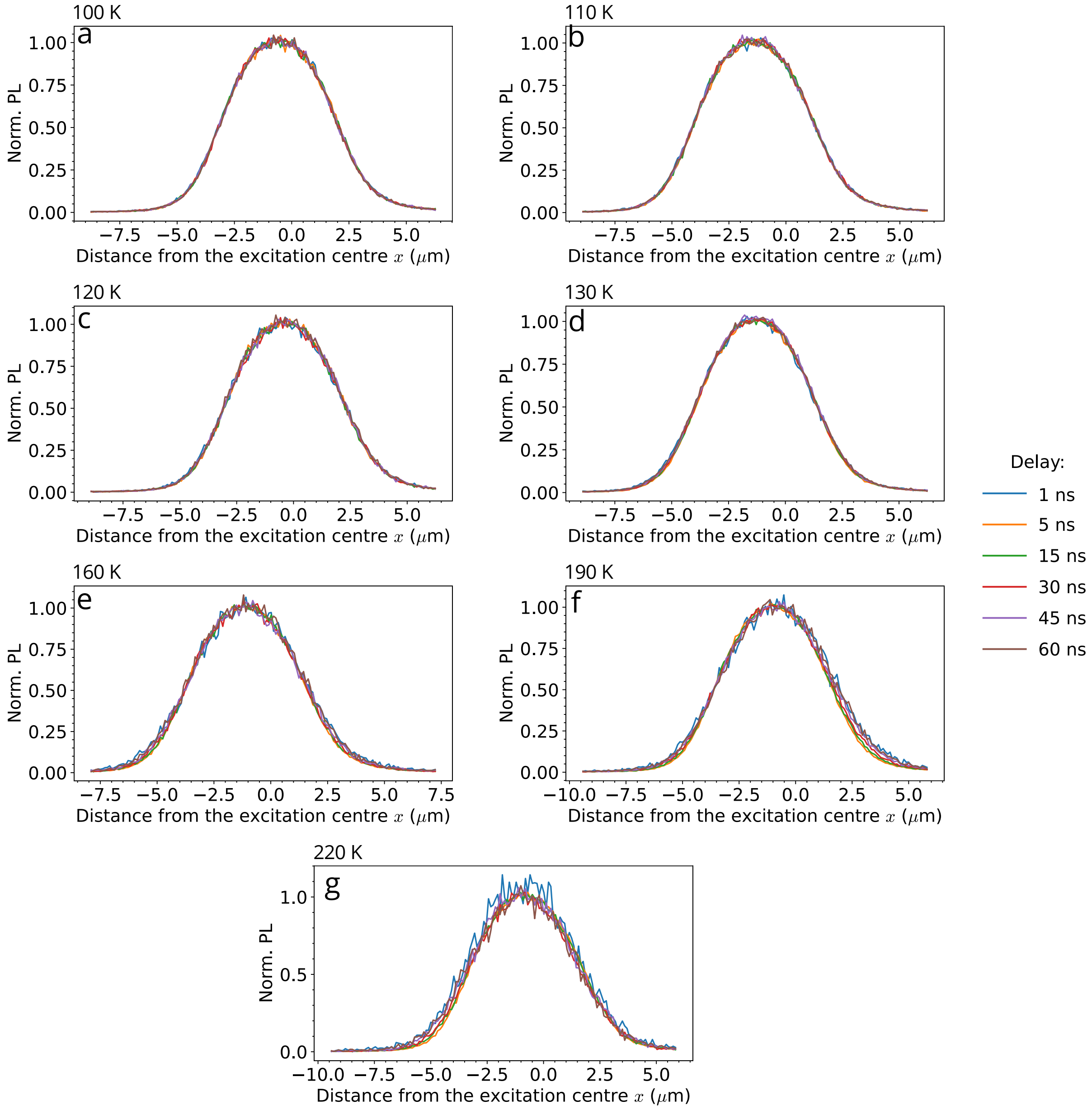

Figure S25: Selected spatial PL profiles for temperatures in the range 100 – 220 K, normalised, at different times after excitation by the laser pulse, localised at x = 0 (centre of the red line in Figure 2a) taken with a 0.4 NA 10x objective lens. While the differences of the lateral width as a function of time seem small on these graphs, they are clearly quantifiable once we apply our Gaussian fitting process.

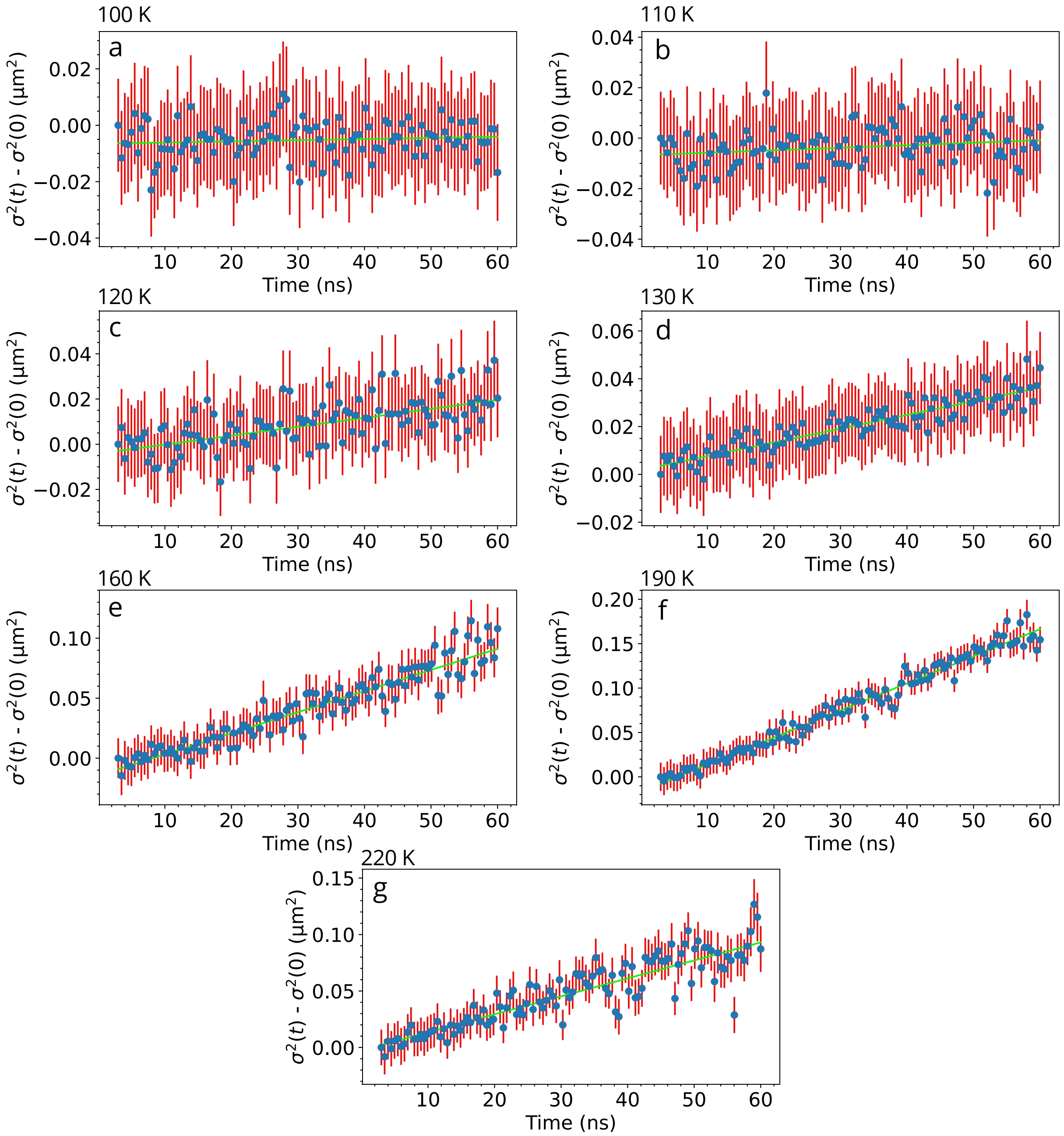

Figure S26: Spreading profiles for temperatures in the range 100 K – 220 K obtained from the n = 4 flake. Data in Figure 4c have been obtained from the fits to Equation 1 shown, green, in the above. Selected Gaussian PL profiles are shown in Figure S25. These figures show a quasi-linear evolution of σ^2 with time which is consistent with a diffusive motion of excitons at these temperatures.

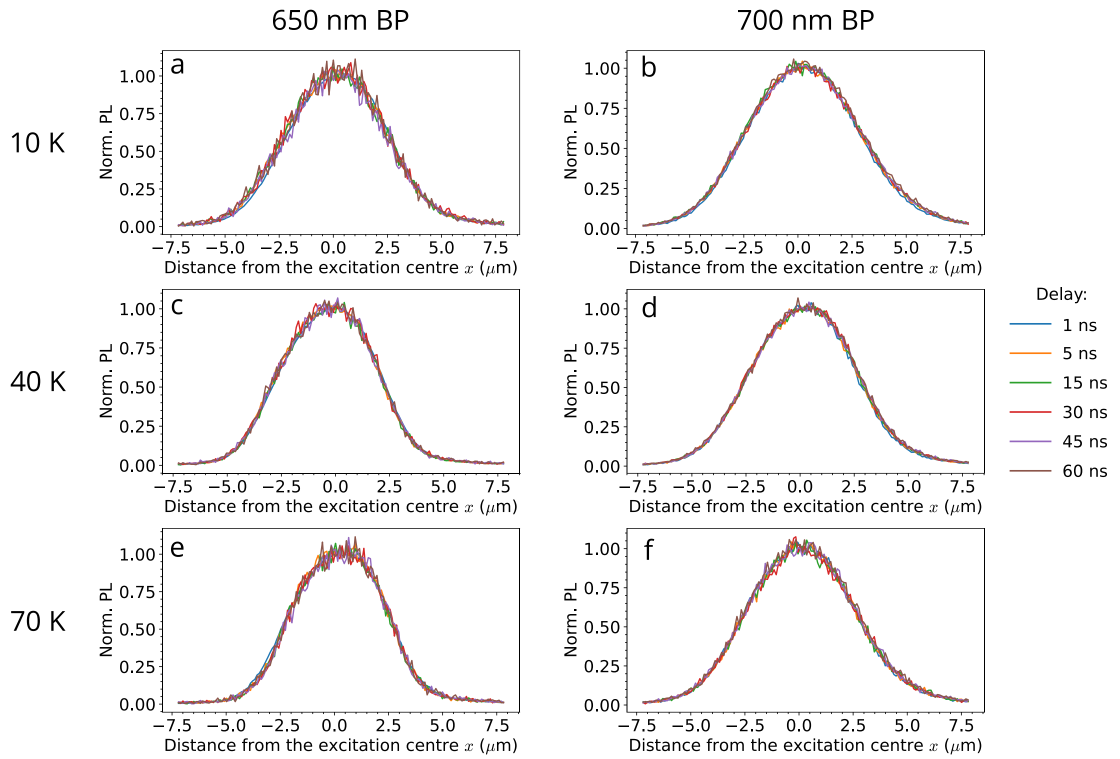

Figure S27: Selected spatial PL profiles for temperatures in the range 10 – 70 K, normalised, at different times after excitation by the laser pulse, localised at x = 0 (centre of the red line in Figure 2a) taken with a 0.4 NA 10x objective lens.

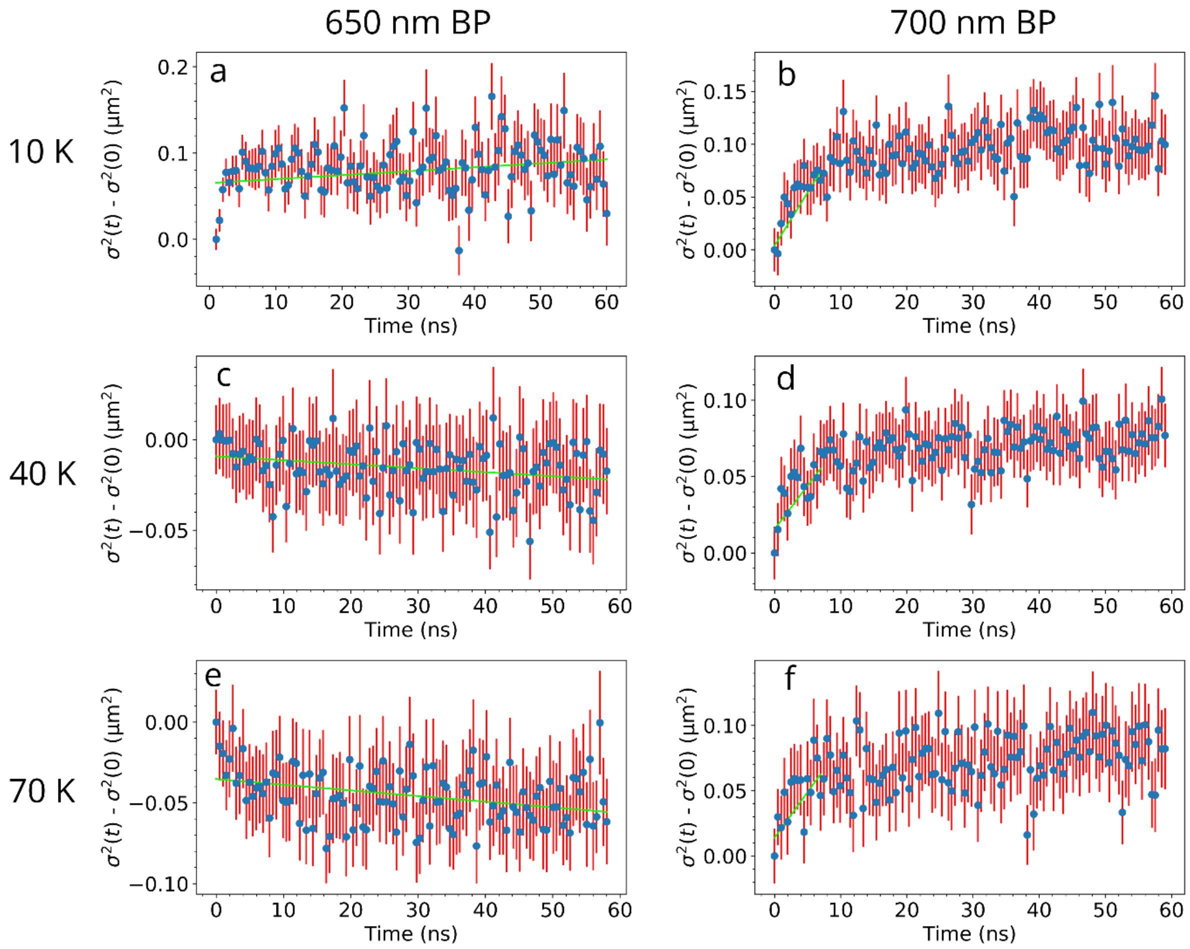

Figure S28: Spreading profiles for temperatures in the range 10 K – 70 K obtained from the n = 4 flake. Band pass filters have been used to enable the PL emission separated into the LE and HE components. Data in Figure 4c have been obtained from the fits shown, green, in the above. Selected Gaussian PL profiles are shown in Figure S27. Figures b, d and f show a fast increase of σ^2 with time, followed by a saturation . As discussed in the main text, this effect is attributed to the efficient drift of excitons at early time, at these temperatures.

XX. Sample degradation

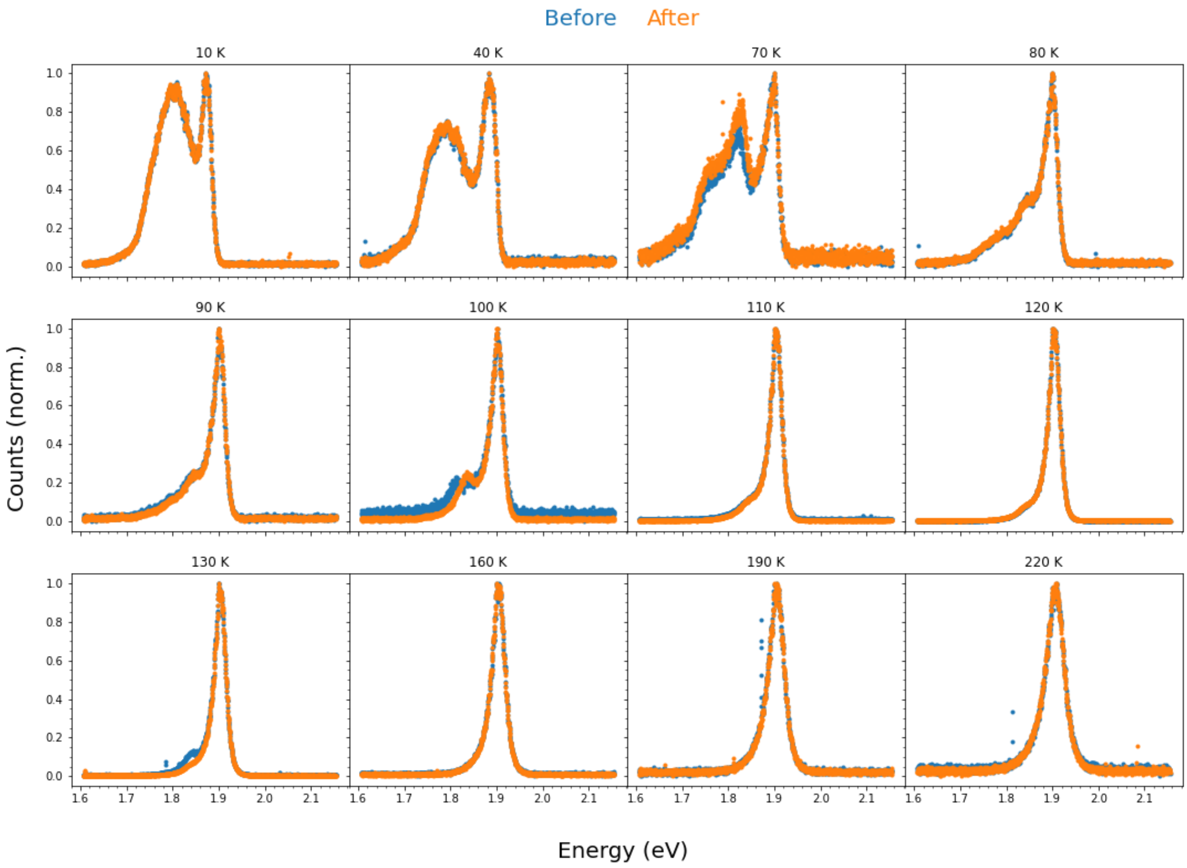

Figure S29: PL spectra of the centre of the region of interest of the n = 4 flake obtained before and after each diffusion measurement at low temperature. Excitation was via a 510 nm laser at a repetition rate of 10 MHz. At low temperatures the samples are less susceptible to degradation due to weekly populated phonon energy levels, as such samples will be able to better accept local heating effects reducing the chances for damage to occur.

XXI. Drift-Diffusion Model:

$$\frac{\partial \mathrm{n}(\vec{\mathrm{r}},\mathrm{t})}{\partial \mathrm{t}} = -kn(\vec{r},\mathrm{t}) + D\nabla^2 n(\vec{r},\mathrm{t}) + \mu\nabla\big(n(\vec{r},t)\nabla \mathrm{u}(\vec{r})\big) \qquad (2),$$

Equation 2 reproduced from the main text.

Model overview:

The model used is based on Equation 2 and experimental data obtained at 10 K. Firstly BP resolved PL confocal images of the flake were obtained. These were individually normalised before being divided by each other to obtain an image showing the distribution of HE and LE domains in the flake, Figure 4d. The PL spectrum obtained at 10 K was normalised to ensure that the integrated intensity of this spectrum equalled to 1. This normalised PL spectrum was subsequently used as a probability density function from which the modelled energetic distribution of the flake was obtained. HE areas in the flake were assigned energies in the range (1.85 – 1.91) eV from the probability density function, while LE areas were assigned energies in the range (1.60 - 1.85) eV. The resulting energetic distribution was used to produce an energetic landscape within the flake used by the model.

Model parameters:

- Δt = 0.0005 ns;
- μ = 1.78 $cm^2\ eV^{-1}\ s^{-1}$;
- D = 0.2495 $cm^2\ s^{-1}$;
- k_{LE} = 0.01 ns^{-1};
- k_{HE} = 0.6 ns^{-1}.

The above parameters are the ones use by the model to produce the modelled data in Figures 5c and 5d. k_{LE} and k_{HE} are the exponential decay rates for the low and high energy regions

respectively obtained from the experimental TRPL data, Figure 5f. μ is the effective mobility for the system which was calculated to be the mobility that would produce an average transfer rate of .5 ns^{-1}, as seen in the TRPL decays of Figure 5f, from the modelled energetic distribution in the flake. D, the diffusion coefficient in the material, remained the only parameter which was manually adjusted to achieve good qualitative agreement with the experimental data.

Figure S30 below displays the outputs from simulations of our proposed model, using the Equation 2 from the main text, for parameters other than those considered in the main text. In particular, panel a corresponds to the purely diffusive case, in which we retrieve the fact that σ^2 is proportional to time, while panel b corresponds to limit in which only drift exist, leading to a sublinear evolution of the σ^2, sometimes referred as the ballistic regime[7].

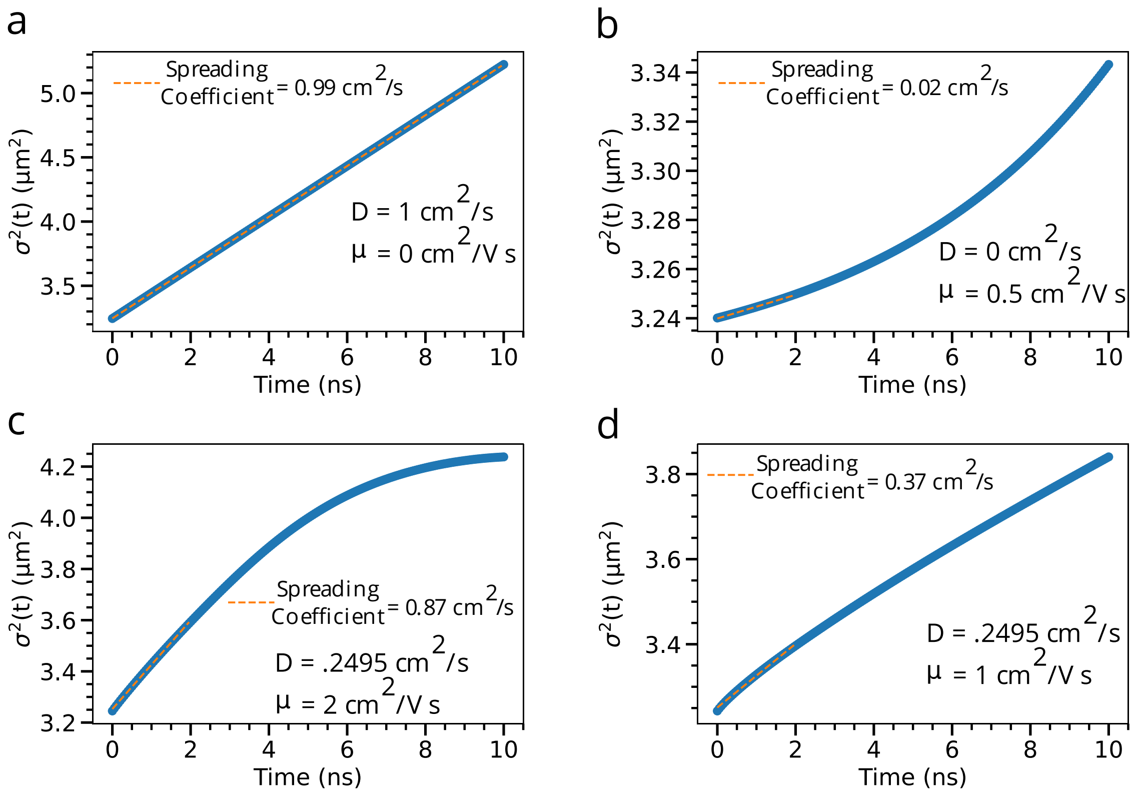

Figure S30: Modelling spreading curves in an RRP flake at low temperature. The used common parameters are detailed above, while any parameters which were altered are shown in the figure. Fits to the initial parts of the data are shown in orange for each resulting simulation.

References.

(1) Leng, K.; Abdelwahab, I.; Verzhbitskiy, I.; Telychko, M.; Chu, L.; Fu, W.; Chi, X.; Guo, N.; Chen, Z.; Chen, Z.; Zhang, C.; Xu, Q.-H.; Lu, J.; Chhowalla, M.; Eda, G.; Loh, K. P. Molecularly Thin Two-Dimensional Hybrid Perovskites with Tunable Optoelectronic Properties Due to Reversible Surface Relaxation. *Nat. Mater.* **2018**, *17* (10), 908–914. DOI: 10.1038/s41563-018-0164-8.

(2) Stoumpos, C. C.; Cao, D. H.; Clark, D. J.; Young, J.; Rondinelli, J. M.; Jang, J. I.; Hupp, J. T.; Kanatzidis, M. G. Ruddlesden–Popper Hybrid Lead Iodide Perovskite 2D Homologous Semiconductors. *Chem. Mater.* **2016**, *28* (8), 2852–2867. DOI: 10.1021/acs.chemmater.6b00847.

(3) Dou, L.; Wong, A. B.; Yu, Y.; Lai, M.; Kornienko, N.; Eaton, S. W.; Fu, A.; Bischak, C. G.; Ma, J.; Ding, T.; Ginsberg, N. S.; Wang, L.-W.; Alivisatos, A. P.; Yang, P. Atomically Thin Two-Dimensional Organic-Inorganic Hybrid Perovskites. *Science (80-.).* **2015**, *349* (6255), 1518–1521. DOI: 10.1126/science.aac7660.

(4) Saha, M. N. LIII. Ionization in the Solar Chromosphere. *London, Edinburgh, Dublin Philos. Mag. J. Sci.* **1920**, *40* (238), 472–488. DOI: 10.1080/14786441008636148.

(5) Blancon, J.-C.; Stier, A. V.; Tsai, H.; Nie, W.; Stoumpos, C. C.; Traoré, B.; Pedesseau, L.; Kepenekian, M.; Katsutani, F.; Noe, G. T.; Kono, J.; Tretiak, S.; Crooker, S. A.; Katan, C.; Kanatzidis, M. G.; Crochet, J. J.; Even, J.; Mohite, A. D. Scaling Law for Excitons in 2D Perovskite Quantum Wells. *Nat. Commun.* **2018**, *9* (1), 2254. DOI: 10.1038/s41467-018-04659-x.

(6) Blancon, J.-C.; Tsai, H.; Nie, W.; Stoumpos, C. C.; Pedesseau, L.; Katan, C.; Kepenekian, M.; Soe, C. M. M.; Appavoo, K.; Sfeir, M. Y.; Tretiak, S.; Ajayan, P. M.;

Kanatzidis, M. G.; Even, J.; Crochet, J. J.; Mohite, A. D. Extremely Efficient Internal Exciton Dissociation through Edge States in Layered 2D Perovskites. *Science (80-.).* **2017**, *355* (6331), 1288–1292. DOI: 10.1126/science.aal4211.

(7) Sung, J.; Schnedermann, C.; Ni, L.; Sadhanala, A.; Chen, R. Y. S.; Cho, C.; Priest, L.; Lim, J. M.; Kim, H.-K.; Monserrat, B.; Kukura, P.; Rao, A. Long-Range Ballistic Propagation of Carriers in Methylammonium Lead Iodide Perovskite Thin Films. *Nat. Phys.* **2020**, *16* (2), 171–176. DOI: 10.1038/s41567-019-0730-2.
